# Re-evaluation of the contribution of *TNFRSF13B* variants to antibody deficiency

**DOI:** 10.70962/jhi.20250016

**Published:** 2025-08-19

**Authors:** Hassan Abolhassani, Andrés Caballero-Oteyza, Mingyu Yang, Michele Proietti, Samaneh Delavari, Patrick Maffucci, Alejandro A. Schäffer, Bertrand Boisson, Jean-Laurent Casanova, Nima Rezaei, Qiang Pan-Hammarström, Charlotte Cunningham-Rundles, Lennart Hammarström, Bodo Grimbacher

**Affiliations:** 1 Division of Immunology, Department of Medical Biochemistry and Biophysics, Karolinska Institutet, Stockholm, Sweden; 2 Research Center for Immunodeficiencies, Children’s Medical Center, Tehran University of Medical Sciences, Tehran, Iran; 3 Institute for Immunodeficiency, Center for Chronic Immunodeficiency, Medical Center, Faculty of Medicine, Albert-Ludwigs-University, Freiburg, Germany; 4 Clinic for Immunology and Rheumatology, Hannover Medical School, Hanover, Germany; 5 RESIST – Cluster of Excellence 2155 to Hanover Medical School, Satellite Center Freiburg, Freiburg, Germany; 6 Division of Allergy and Clinical Immunology, Departments of Medicine and Pediatrics, Icahn School of Medicine at Mount Sinai, New York, NY, USA; 7 Center for Cancer Research, National Cancer Institute, National Institutes of Health, Bethesda, MD, USA; 8 St. Giles Laboratory of Human Genetics of Infectious Diseases, Rockefeller Branch, The Rockefeller University, New York, NY, USA; 9 Laboratory of Human Genetics of Infectious Diseases, Necker Branch, INSERM U1163, Necker Hospital for Sick Children, Paris, France; 10 Université Paris Cité, Imagine Institute, Paris, France; 11 Department of Pediatrics, Necker Hospital for Sick Children, Paris, France; 12 Howard Hughes Medical Institute, New York, NY, USA; 13 Clinic of Rheumatology and Clinical Immunology, Center for Chronic Immunodeficiency (CCI), Medical Center, Faculty of Medicine, Albert-Ludwigs-University of Freiburg, Freiburg, Germany; 14 DZIF – German Center for Infection Research, Satellite Center Freiburg, Freiburg, Germany; 15 CIBSS – Centre for Integrative Biological Signalling Studies, Albert-Ludwigs University, Freiburg, Germany

## Abstract

Predominantly antibody deficiency (PAD) is the most prevalent form of human inborn errors of immunity (IEI). PAD is characterized by recurrent bacterial infections, immune dysregulation, and impaired immunoglobulin production. A monogenic cause of PAD can be identified in about 20% of cases. Approximately 10% of patients carry heterozygous mutations in the tumor necrosis factor receptor superfamily member 13B gene (*TNFRSF13B*), encoding the B cell surface protein TACI. Heterozygous variants in *TNFRSF13B* are not sufficient to cause PAD, as ∼1% of the healthy population carries one of these variants. To identify additional genetic contributors to the immune defect in these individuals, we examined the exomes of 161 PAD patients with rare-damaging variants in *TNFRSF13B*. We identified (1) biallelic mutations in *TNFRSF13B*, (2) the HLA class II marker (DPA1*03), and (3) multiple single nucleotide polymorphisms in known B cell-related genes as additional genetic risk factors. Moreover, pathogenic mutations in other known IEI genes were presented in 16% of patients with heterozygous *TNFRSF13B* variants.

## Teaser

The presence of a second TNFRSF13B mutation, HLA class II markers, or multiple single nucleotide variants in patients helps to explain the incomplete penetrance of immunodeficiency in carriers of TNFRSF13B mutations.

## Graphical Abstract

**Figure:**
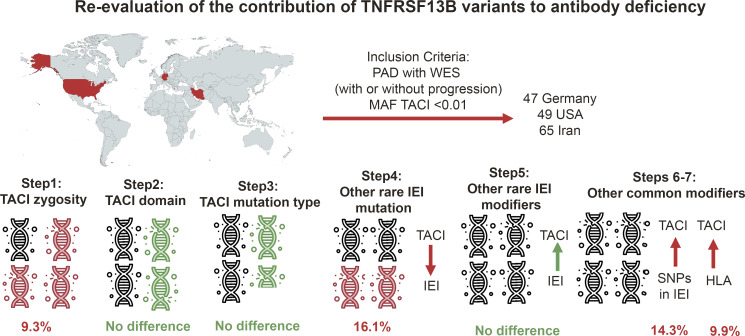

## Introduction

The transmembrane (TM) activator and calcium modulator and cyclophilin ligand interactor (TACI, encoded by tumor necrosis factor receptor superfamily member 13B gene [*TNFRSF13B*]) is a member of the TNF receptor superfamily expressed on B cells in secondary lymphoid organs and is important for peripheral B cell homeostasis (1, 2). However, how TACI and its associated downstream molecules fine tune B cell biology remains incompletely understood. TACI mediates immunoglobulin class switch recombination, differentiation and survival of plasma cells, and T-independent responses to polysaccharide antigens (3, 4, 5). TACI also acts as an immunoregulator involved in central B cell tolerance (6, 7). Variants in the gene encoding TACI (*TNFRSF13B*) have been identified since 2005 in patients with predominantly antibody deficiency (PAD) employing a candidate gene approach based on single-gene knockout mice (1, 8, 9). Although *TNFRSF13B* mutations were initially thought to be fully penetrant, which may be the case for individuals with biallelic mutations, it is now known that monoallelic TACI variants are by themselves not enough to cause a symptomatic phenotype (10, 11, 12, 13). Although experimental studies *in vitro* suggest haploinsufficiency or a dominant-negative impact of these variants as a tentative mechanism of action, these hypotheses do not explain the fact that ∼1% of the healthy population also carries one of the disease-associated mutations. Moreover, the observation that the risk of developing PAD in any given offspring in a family with a variant in *TNFRSF13B* and a diagnosed patient with PAD is close to 25% (i.e., 50% of *TNFRSF13B* mutation carriers) suggests the presence of additional modifying genes in patients (4, 14, 15, 16, 17, 18, 19, 20, 21, 22, 23, 24, 25, 26, 27).

Two main PADs have been linked with *TNFRSF13B* mutations: common variable immunodeficiency (CVID) and selective immunoglobulin A deficiency (sIgAD) (6, 28, 29, 30). Although more than 30 variants have been reported in TACI-deficient patients, no clear genotype–phenotype association has been shown to date. Both biallelic (homozygous p.S144X, homozygous p.C104R, homozygous p.A181E, compound heterozygous p.C104R/p.L69fsX11, compound heterozygous p.C104R/p.S194X, compound heterozygous L171R/A181E, compound heterozygous p.C193X/p.Y102X, and compound heterozygous p.C193X/p.C104R) and monoallelic (heterozygous p.R9X, p.Q17fsX, p.M31fsX, p.C34X, p.S33fsX, p.W40R, p.D41H, p.C66X, p.L69fsX11, p.R72H, p.G76fsX, p.Y79C, p.I87N, p.C100fsX, p.Y102X, p.C104R, p.C104Y, p.E117fsX, p.R119fsX, p.Q121X, p.S144X, p.Y164X, p.T166fsX, p.L171R, p.C172Y, p.A181E, p.C184X, p.K188M, p.R189K, p.C193X, p.S194X, p.R202H, p.V220A, p.E236X, p.V246F, and p.P251L) *TNFRSF13B* variants have been reported (31, 32, 33). Specific heterozygous pathogenic splicing mutations (c.61+1G>T, c.61+1G>A, c.61+2T>A, c.61+2T>C, c.62-1G>A, and c.62-2A>G, c.200-2A>G) were also reported in the ClinVar database associated with antibody deficiency (33). The only observation about different genotypes is that, paradoxically, biallelic mutations seem to always confer a phenotype, but that the phenotype is somewhat milder than the phenotype in patients who come to clinical attention with heterozygous *TNFRSF13B* mutations (12, 27). Of note, *TNFRSF13B* has multiple transcripts, and the most common heterozygous variants, p.C104R and p.A181E, may not disturb membrane receptor formation (longer encoded transcript of the gene) but can impact the intracellular (IC) compartment (smaller encoded transcript in the marginal zone, isotype-switched B cells, and plasmablasts) (34). Still to be dissected is the control of expression of the two *TNFRSF13B* isoforms, only one of which is fully functional (34, 35). Population studies have shown that heterozygous (but not biallelic) variants can be identified at a prevalence of ∼1% within population databases and specific regional genetic populations (12, 17, 18, 19, 36, 37). This suggests that the phenotypic expression of these *TNFRSF13B* mutations as PAD is affected by additional genetic or environmental factors (38, 39, 40).

Depending on the number of genetic modifier loci, the analysis of a large sample of *TNFRSF13B* mutation carriers will be needed to determine whether a specific combination of genetic markers (including the *TNFRSF13B* mutation) is associated with a specific phenotype or predisposition to the common features of PAD, including infections, autoimmunity, lymphoproliferation, or malignancy. To achieve this goal, we evaluated the exomes of 161 *TNFRSF13B* mutation carriers and investigated their medical records regarding clinical manifestations, immunologic phenotypes, genetic modifiers, and survival outcomes.

## Results

### Clinical features of the cohort of PAD patients with TNFRSF13B mutations

A total of 161 patients (52.8% females) with confirmed *TNFRSF13B* mutations were recruited from three clinical centers into this study between 2019 and 2023. The median (IQR) age of patients was 36.5 (7.5–63.0) years at the time of the study and 4.0 (1.0–10.5) years at the onset of symptoms. The diagnostic delay was 7.0 (5.0–35.0) years. Consanguinity was present in 27 (16.7%, all from the Iranian cohort) patients. All patients suffered from upper respiratory tract infections, and 45.9% of patients had lymphoproliferative manifestations. Other frequent clinical presentations of immunodeficiency in patients with *TNFRSF13B* mutations were recurrent autoimmunity (38.5%), lower respiratory infections (30.4%), enteropathy (26.6%), atopic manifestations (16.1%), and cancer (9.9%). Patients were diagnosed with various forms of antibody defect, including CVID (*n* = 134, 83.2%), selective IgG deficiency (*n* = 18, 11.1%), sIgAD (*n* = 7, 4.3%), and specific antibody deficiency (*n* = 2, 1.2%). Progressive forms of immunodeficiency were observed during the average 12-year follow-up period in 21 patients (13.0%), mainly with the escalation of PAD to a combined immunodeficiency (*n* = 12, 7.4%) and sIgAD to CVID (*n* = 9, 5.6%). Demographic, clinical, and immunological data of patients are summarized in Tables 1 and 2.

**Table 1.** Clinical characteristics of 161 TACI-deficient patients at last follow-up

Parameters	Total (*n* = 161)	Monoallelic (*n* = 143)	Biallelic (*n* = 18)	P value	Missense (*n* = 153)^a^	Non-missense (*n* = 22)^b^	P value	EC (*n* = 123)^c^	IC (*n* = 16)^d^	TM (*n* = 36)^e^	P value
Male/Female	76/85	69/74	7/11	0.45	65/74	11/11	0.77	49/60	8/8	19/17	0.69
Mortality, number (%)	6 (3.7)	4 (2.8)	2 (11.1)	0.07	5 (3.2)	1 (4.5)	0.55	4 (3.3)	1 (6.3)	1 (2.8)	0.80
Age at time of study, median (IQR), and years	36.5 (7.5–63.0)	35.5 (18–51)	56 (39–60)	0.03*	38 (24.5–54)	20 (53–14.5)	0.20	35.5 (18.2–55)	38 (17–53)	38.5 30.5–48)	0.49
Age of onset, median (IQR), years	4.0 (1.0–10.5)	2.1 (1–7)	24 (13–34)	0.01*	2.5 (1–8.7)	1 (0.5–2.0)	0.76	2 (1–8.2)	1 (0.5–3)	3 (1.5–1.5)	0.06
Diagnostic delay, median (IQR), and years	7.0 (5.0–35.0)	7 (4–32)	14 (10–39)	<0.001*	7.5 (5–34)	5 (3.5–9)	0.55	7.0 (4.5–34)	5.0 3.5–30)	9 (6.5–37)	0.43
Clinical CVID diagnosis, number (%)	134 (83.2)	119 (83.2)	15 (83.3)	0.55	117 (76.5)	17 (77.3)	0.93	94 (76.4)	12 (75)	28 (77.8)	0.97
Progressive form of PAD, number (%)	21 (13.0)	14 (9.8)	4 (22.2)	0.11	17 (11.1)	4 (18.2)	0.33	14 (11.4)	2 (12.5)	4 (11.1)	0.98
Parental consanguinity, number (%)	27 (16.7)	24 (16.8)	3 (16.7)	0.99	23 (15)	4 (18.2)	0.70	18 (14.6)	1 (6.3)	8 (22.2)	0.49
URI, number (%)	161 (100)	143 (100)	18 (100)	1.0	153 (100)	22 (100)	1.0	123 (100)	16 (100)	36 (100)	1.0
Otitis, number (%)	121 (75.2)	109 (76.2)	12 (66.7)	0.37	107 (69.9)	14 (63.6)	0.54	84 (68.3)	10 (62.5)	27 (75)	0.62
Sinusitis, number (%)	81 (50.3)	66 (46.2)	15 (83.3)	0.002*	68 (44.4)	13 (59.1)	0.19	59 (48)	7 (43.8)	15 (41.7)	0.78
LRI, number (%)	49 (30.4)	43 (30.0)	6 (33.3)	0.77	45 (29.4)	4 (18.1)	0.27	34 (27.6)	5 (31.3)	10 (27.7)	0.95
Pneumonia, number (%)	29 (18.0)	25 (17.4)	4 (22.2)	0.62	28 (18.3)	1 (4.5)	0.10	22 (17.8)	3 (18.8)	4 (11.1)	0.61
Bronchiectasis, number (%)	21 (13)	15 (10.5)	6 (33.3)	0.006*	18 (11.8)	3 (13.6)	0.80	15 (12.2)	3 (18.8)	3 (8.3)	0.56
Autoimmunity, number (%)	62 (38.5)	56 (39.1)	6 (33.3)	0.63	54 (35.2)	8 (36.4)	0.92	45 (36.5)	5 (31.3)	12 (33.3)	0.87
Lymphoproliferative, number (%)	74 (45.9)	61 (42.6)	13 (72.2)	0.03*	63 (41.1)	11 (50)	0.43	50 (40.6)	7 (43.8)	17 (48.2)	0.77
Allergy/atopy, number (%)	26 (16.1)	21 (14.6)	5 (27.8)	0.15	21 (13.7)	5 (22.7)	0.26	20 (16.2)	0 (0)	6 (16.6)	0.16
Enteropathy, number (%)	38 (26.6)	33 (23.0)	5 (27.8)	0.65	31 (20.2)	7 (31.8)	0.21	32 (26.0)	0 (0)	6 (16.7)	0.02*
Malignancy, number (%)	16 (9.9)	14 (9.7)	2 (11.1)	0.85	10 (6.3)	6 (27.2)	0.001*	11 (8.9)	0 (0)	5 (13.8)	0.31

EC, extracellular domain; IC, intracellular domain; TM, transmembrane domain; URI, upper respiratory infections; LRI, lower respiratory infections; IQR, interquartile range.

EC (positions 1–165), TM (positions 166–186), and IC (positions 187–293).

*Significant P value <0.05.

a 153 patients with missense variants, including 130 with one variant, 19 with two variants in a compound heterozygous pattern and 4 with the same variant repeated in a homozygous pattern.

b 22 patients with non-missense variants, 8 in a compound heterozygous pattern and 1 in a homozygous pattern.

c 123 patients with TACI EC defects, 17 in a compound heterozygous pattern and 4 in a homozygous pattern.

d 16 patients with TACI IC defects, 5 in a compound heterozygous pattern.

e 36 patients with TACI TM defects, 5 in a compound heterozygous pattern and 4 in a homozygous pattern.

**Table 2.** Immunologic profile of 161 TACI-deficient patients at the time of PAD diagnosis

Parameters	Total (*n* = 161)	Monoallelic (*n* = 143)	Biallelic (*n* = 18)	P value	Missense (*n* = 153)^a^	Non-missense (*n* = 22)^b^	P value	EC (*n* = 123)^c^	IC (*n* = 16)^d^	TM (*n* = 36)^e^	P value
WBC count Median (IQR), cells/μl	6,690 (5,300–8,500)	4,700 (3,915–5,900)	6,780 (5,600–8,500)	0.22	6,690 (5,400–8,400)	6,850 (4,850–8,525)	0.5	7,000 (5,450–8,900)	6,090 (5,025–7,545)	6,375 (5,590-7,052.5)	0.37
Lymphocyte absolute count Median (IQR), cells/μl	1,960 (1,520–2,970)	1,023 (970–1,319.7)	1,977 (1,560–3,010)	0.14	1,977 (1,572–2,970)	1,525 (1,185.7–2,432.5)	0.36	2,087 (1,545–3,055)	1,800 (1,255–2,787.5)	1,750 (1,527.5–2,007.5)	0.63
CD3 + T cells absolute count Median (IQR), cells/μl	1,530 (1,069.5–1,951.5)	954 (954–1,157)	1,546 (1,096.5–1,957.2)	0.31	1,550 (1,110–1,951.5)	1,154 (995.2–1,620)	0.28	1,750 (1,120–1,970)	1,479.5 (9,67.7–1,995)	1,342.5 (1,074.5–1,455.7)	0.70
CD19 + B cells absolute count Median (IQR), cells/μl	302 (119–425)	45 (45–372.5)	308 (120–424)	0.62	314 (133.5–416.5)	205 (99.75–531.2)	0.75	302 (118–427)	120 (72–359.2)	332 (155–387.5)	0.99
CD27-IgM+ naïve B cell Median (IQR), % of total B cells^f^	76.5 (59.5–87.7)	75 (60.2–89)	81.2 (58.5–90)	0.32	74.2 (59.5–87.7)	81.2 (61.7–92)	0.21	76.2 (59–88.5)	78.5 (60.5–90.3)	75.2 (57.2–84)	0.66
CD27+IgM- switched memory B cell Median (IQR), % of total B cells^f^	1.2 (0.5–6.2)	1.0 (0.2–4.4)	4.0 (0.5–15.2)	0.17	1.5 (0.5–6.2)	0.7 (0.2–9.5)	0.10	1.2 (0.7–5.7)	0.7 (0.2–7.5)	1.7 (0.5–7)	0.73
IgM Median (IQR), mg/dl	25 (18.2–42)	25.5 (24.2–80.7)	24 (18–41.7)	0.17	26 (20–42)	19 (13–32)	0.51	25 (17–40)	26 (23–55)	27.5 (20–42)	0.60
IgG Median (IQR), mg/dl	309 (143.2–465.2)	347.5 (175–433.5)	294.5 (125.25–465.2)	0.98	308 (141–460)	302 (175–583)	0.86	278 (120–467)	405 (310–480)	318 (187–393)	0.43
IgA Median (IQR), mg/dl	16.5 (7–30)	27.5 (18–69)	15 (7–26)	0.04*	19 (7–32)	12 (8–20)	0.34	17 (6–24)	20 (12–26)	15.5 (9.5–51.5)	0.45
IgE Median (IQR), IU/ml	2 (0–12)	4 (4–8)	2 (0–12.5)	0.94	2 (0–12.5)	4 (1.5–10)	0.79	2.5 (0–12)	2 (2–4)	2 (0.5–14.5)	0.76

EC, extracellular domain; IC, intracellular domain; TM, transmembrane domain; IQR, interquartile range.

*Significant P value <0.05.

a 153 patients with missense variants, 19 in a compound heterozygous pattern and 4 in a homozygous pattern.

b 22 patients with non-missense variants, 8 in a compound heterozygous pattern and 1 in a homozygous pattern.

c 123 patients with TACI EC defects, 17 in a compound heterozygous pattern and 4 in a homozygous pattern.

d 16 patients with TACI IC defects, 5 in a compound heterozygous pattern.

e 36 patients with TACI TM defects, 5 in a compound heterozygous pattern and 4 in a homozygous pattern.

f Data on B cell subset were only available for 134 cases.

### Diagnostic genetic results of the *TNFRSF13B *gene

**Figure 1.**
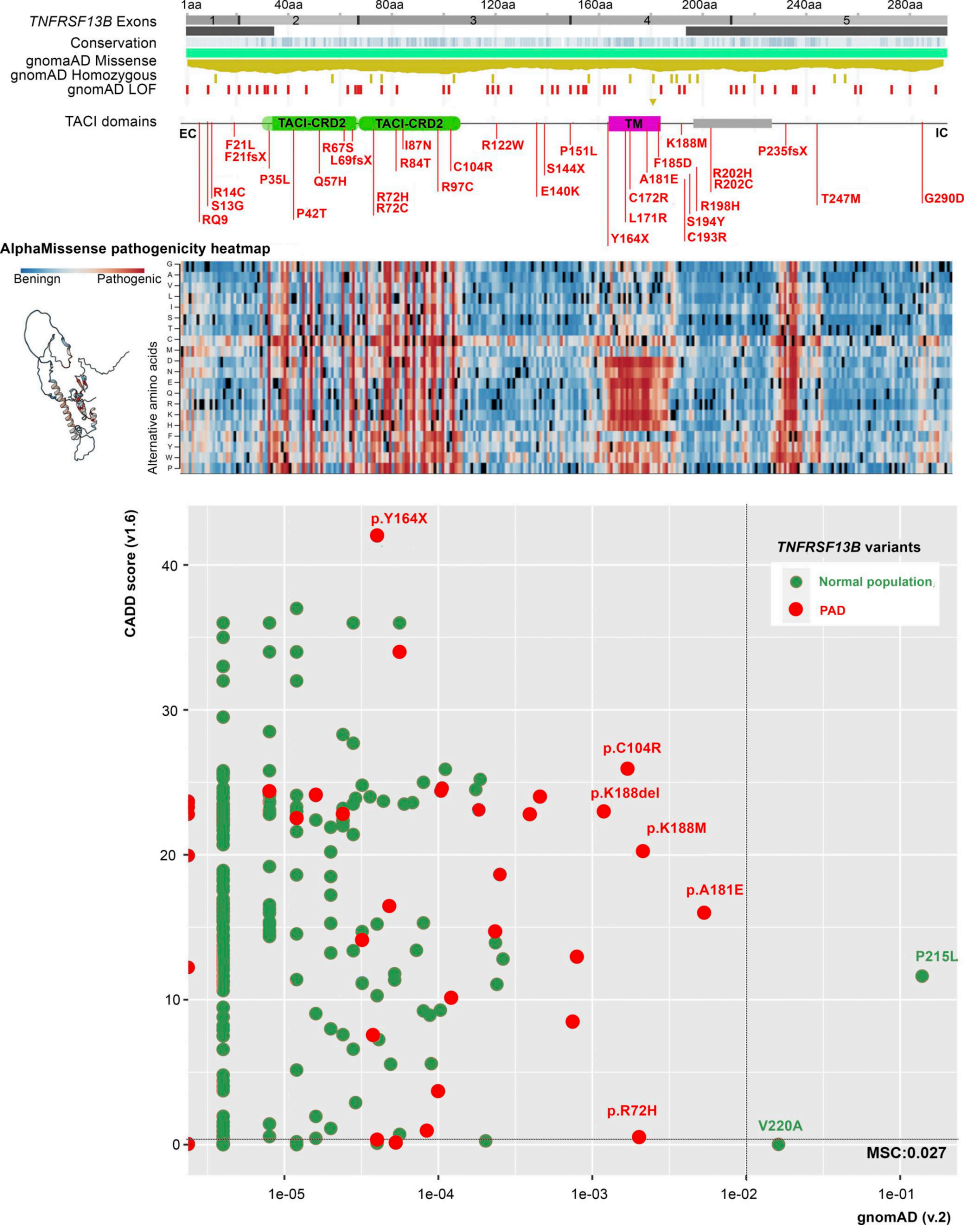
Spectrum of 38 unique *TNFRSF13B* variants identified in 161 PAD patients, showing the exonic locations, affected protein domains, AlphaMissense pathogenicity heatmap, population allele frequency, and CADD predicted damaging score compared with MSC and other variants observed in normal populations based on Genome Aggregation Database (gnomAD).

**Figure S1.**
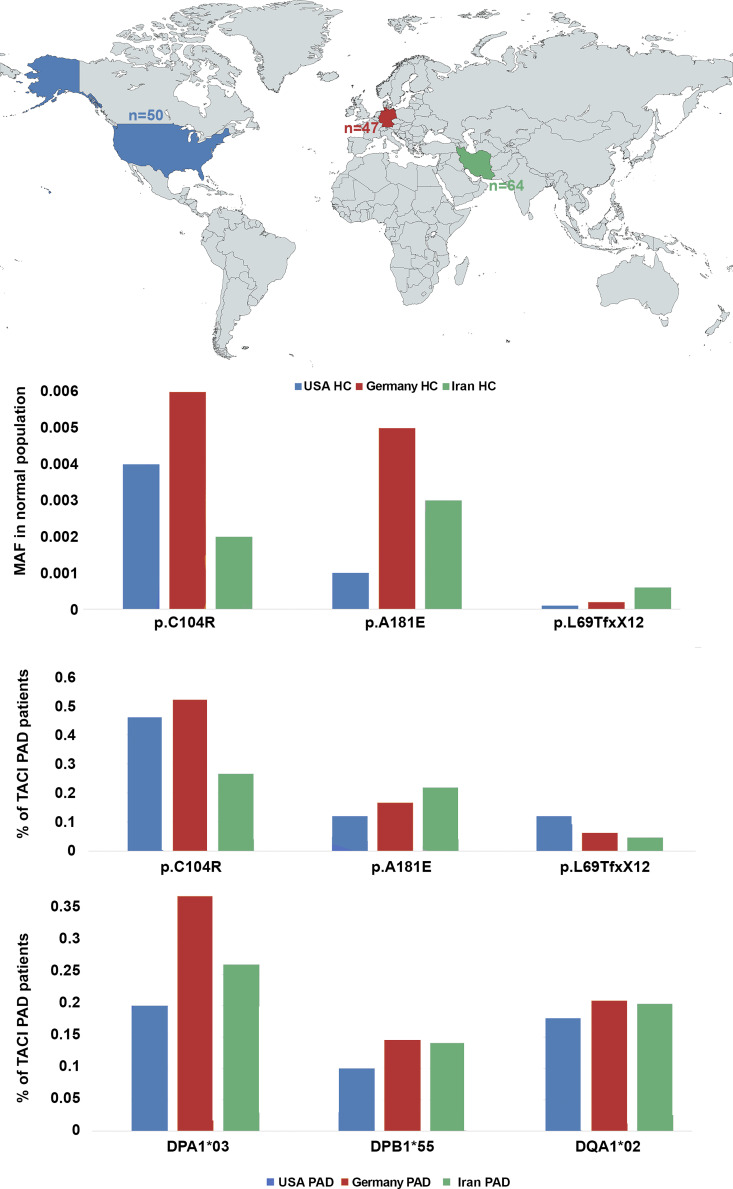
Frequencies of most common *TNFRSF13B* variants and significantly associated HLA alleles in PAD patients from three different cohorts.

Molecular diagnosis was conducted using whole-exome sequencing (WES) in all the 161 patients who participated in this study. The detailed genetic analysis results are provided in Table 3. Genetic reanalysis confirmed rare *TNFRSF13B* variants with combined annotation-dependent depletion (CADD) scores above the mutation significance cutoff (MSC) for all patients. 88.8% carried a single allele variant (*n* = 143), and 12.2% (*n* = 18) had two heterozygous or one homozygous mutations, accounting for 7.8% (*n* = 13) and 3.5% (*n* = 5) of patients, respectively. 38 unique variants were found in our study, 8 of which have not been reported previously in healthy individuals, with the majority of these located in the extracellular (EC) domain and highly conserved regions of the protein predicted damaging by AlphaMissense pathogenicity heatmap (Table 3 and Fig. 1). The most frequent type of mutation was a missense mutation, which was observed in 93.7% of patients. Frameshift and stop-gain mutations were observed in 8.6% and 3.1% of patients, respectively; the percentages added to more than 100% because of the compound heterozygous mutations, which may be of different types. Other types of mutations were seen in only three patients, including splice site mutations (*n* = 2) and an in-frame deletion (*n* = 1). The most common *TNFRSF13B* mutations in this cohort were p.C104R (*n* = 65, 40.3% of patients), p.A181E (*n* = 26, 16.1%), and p.L69TfsX12 (*n* = 12, 7.4%). Of note, seven mutations were only present as part of a biallelic combination, including missense (p.P35L, p.F185C, p.S194Y, and p.C193R), stop-gain (p.S144X and p.Y164X), and splice site (c.61+1 G>T) mutations. None of the frequent mutations were significantly over- or underrepresented among the patients from any of the three study sites, and the mutation frequencies are only slightly different from observed allele frequency in normal populations (Fig. S1).

**Table 3.** TNFRSF13B genetic mutations of 161 PAD patients based on gnomAD MAF < 0.01

TACI variants	Total	Mono allelic	Biallelic	Type	Domain	CADD	gnomAD, MAF	Top MAF in different populations
p.Cys104Arg	65	43	22	Missense	EC	25.7	0.003400	0.005 in European non-Finnish
p.Ala181Glu	26	24	2	Missense	TM	16.01	0.005538	0.024 in European Finnish^b^
p.Leu69ThrfsX12	12	8	4	Frameshift	EC	22.8	0.0003949	0.005 in Ashkenazi Jewish
p.Arg72His	9	9	0	Missense	EC	0.5	0.002003	0.003 in European non-Finnish
p.Ile87Asn	7	6	1	Missense	EC	24	0.0004237	0.0007 in European non-Finnish
p.Cys172Tyr	5	5	0	Missense	TM	23.1	0.0001810	0.0004 in Ashkenazi Jewish
p.Arg122Trp	4	4	0	Missense	EC	18.64	0.0002321	0.0003 in European non-Finnish
p.Gln57His	4	4	0	Missense	EC	14.71	0.0002432	0.0007 in South Asian
p.Cys193Arg	3	0	3	Missense	IC	20.1	0.00009241	0.0001 in European non-Finnish
p.Lys188Met	3	3	0	Missense	IC	20.0	0.0046	0.013 in South Asian^b^
p.Ser144X	3	0	3	Stop-gain	EC	34	0.00005718	0.0001 in European non-Finnish
p.Arg202His	2	2	0	Missense	IC	23	0.0007783	0.003 in Ashkenazi Jewish
p.Arg72Cys	2	2	0	Missense	EC	12.96	0.00006	0.0001 in South Asian
p.Leu171Arg	2	2	0	Missense	TM	10.15	0.0001034	0.0001 in European non-Finnish
p.Phe185Cys	2	0	2	Missense	TM	24.4	0.000004095	0.000008 in European non-Finnish
p.Pro151Leu	2	2	0	Missense	EC	0.35	0.0001190	0.0001 in Latino
p.Pro42Thr	2	2	0	Missense	EC	22.5	0.00004085	0.0002 in South Asian
p.Ser194Tyr	2	0	2	Missense	IC	24.4	0.00001479	0.00003 in European non-Finnish
c.61+1 G>T	1	0	1	Splicing	EC	32	0^c^	-
c.61+5 G>A	1	1	0	Splicing	EC	8.46	0^c^	-
p.Arg14Cys	1	1	0	Missense	EC	3.69	0.00004495	0.00008 in Latino
p.Arg198His	1	1	0	Missense	IC	24.6	0.0006888	0.005 in South Asian
p.Arg202Cys	1	1	0	Missense	IC	0.12	0.0001260	0.001 in African
p.Arg67LysfsX23	1	1	0	Frameshift	EC	16.46	0^c^	-
p.Arg67Ser	1	1	0	Missense	EC	42	0^c^	-
p.Arg84Thr	1	1	0	Missense	EC	7.541	0.00003316	0.0004 I in East Asian
p.Arg9Gln	1	1	0	Missense	EC	14.13	0.00003269	0.0001 in Latino
p.Glu140Lys	1	1	0	Missense	EC	11.16	0.00002450	0.0001 in Latino
p.Gly290Asp	1	1	0	Missense	IC	22.8	0.00003770	0.0006 in East Asian
p.Lys188del	1	1	0	Deletion	IC	24.1	0.001570	0.013 in South Asian^b^
p.Phe21Leu	1	1	0	Missense	EC	23.3	0^c^	-
p.Phe21SerfsX2	1	1	0	Frameshift	EC	19.95	0^c^	-
p.Pro235ArgfsX169	1	1	0	Frameshift	IC	12.23	0.0001058	0.001 in East Asian
p.Pro35Leu	1	0	1	Missense	EC	23.7	0^c^	-
p.Pro97Arg	1	1	0	Missense	EC	22.8	0.00001634	0.00003 in European non-Finnish
p.Ser13Gly	1	1	0	Missense	EC	0.022^a^	0^c^	-
p.Thr247Met	1	1	0	Missense	IC	0.062	0.00004979	0.0005 in East Asian
p.Tyr164X	1	0	1	Stop-gain	TM	23.6	0.00004435	0.00009 in European non-Finnish

EC, extracellular domain; IC, intracellular domain; TM, transmembrane domain; gnomAD, Genome Aggregation Database.

a Below MSC-CADD (V1.6) threshold for TACI gene: 0.027.

b Specific allele in population with MAF > 0.01.

c Variants never observed in normal population.

### Clinical and immunologic manifestations in different groups of TNFRSF13B mutations

Patients with biallelic *TNFRSF13B* mutations had a much later onset (24.0 vs. 2.1 years, P = 0.01) and longer diagnostic delay (14.0 vs. 6.8 years, P < 0.001) compared with monoallelic patients. Patients with a biallelic mutation were older at the time of our study and presented with higher rates of sinusitis (83.3% vs 46.2%, P = 0.002), bronchiectasis (33.3% vs 10.5%, P = 0.006), and lymphoproliferative disorders (72.2% vs 42.6%, P = 0.03) compared with patients with single *TNFRSF13B* mutations (Table 1). Moreover, patients with the non-missense deleterious mutations had a higher susceptibility to cancer development (mainly due to lymphoma and carcinomas) in comparison to cases with missense *TNFRSF13B* mutations (27.2% vs. 7%, P = 0.001). Although the affected domains of the protein had almost similar demographic and clinical presentations, individuals with a localized mutation in the EC domain had a higher frequency of enteropathy as compared with patients with mutations in the IC domain (26.0% vs. 0%, P = 0.02). The reduction of serum immunoglobulin levels in patients with biallelic mutations was slightly more prominent compared with monoallelic cases; in particular, IgA levels were significantly different between these two groups, 15 (7–26) mg/dl versus 27.5 (18–69) mg/dl (P = 0.04). Immunoglobulin profiling also indicated a less severe reduction in patients with missense and IC domain mutations, but the difference was not significant. Moreover, leukocyte counts and frequencies of lymphocyte subsets were similar between subjects with different groups of mutations (Tables 1 and 2).

### Other genetic mutations and polymorphism in TNFRSF13B-mutated patients

First, we focused on the other rare variants in known inborn errors of immunity (IEI) genes. In Table S1, we list all rare non-synonymous exonic variants/mutations of IEI genes (as defined by the 2024 IUIS classification) identified within the TACI cohort; Table S2 presented pathogenic/likely pathogenic and variants of unknown significance and classified them into three categories: B cell defects, T cell defects, and bone marrow failure. Then, all rare modifier IEI variants were extracted from the WES data and compared with 1,241 in-house unsolved PAD patients. The frequency of both complete mutations (fitting both the Mendelian inheritance and American College of Medical Genetics and Genomics, ACMG, criteria) or modifiers (the remaining variants) was not significantly different between the TACI cohort and the unsolved PAD cohort. Of note, the two genes with impact on B cells, *DKC1* (augmenting telomerase activity required for B cell continuous cellular proliferation) and *CXCR4* (orchestrating of B cell migration and homing between bone marrow and periphery), were more frequently mutated in TACI patients compared with other PAD patients (Table S3). Notably, the patients with mutations in *CXCR4* all had their mutations in the homozygous state and had normal absolute neutrophil counts, even though other mutations in *CXCR4* are known to cause a neutropenia syndrome (https://www.omim.org/entry/193670). Some specific TACI patients also carried known pathogenic mutations (based on ACMG criteria) in other IEI genes, including *BTK*, *NFKB1*, *RAG1*, *RAG2*, *CTLA4*, *TCF3*, *STAT3*, *IKZF2*, *BACH2*, *NHEJ1*, *CD27*, *AK2*, *ATM*, and *SAMD9* (Table S2). Interestingly, all these pathogenic mutations in other IEI genes, mostly considered to have complete penetrance, were identified exclusively in patients with monoallelic TACI mutations, but there was no significant association between presence/absence of a mutation in a second IEI gene and either the type of heterozygous *TNFRSF13B* mutation (missense versus non-missense variant) or the location of the heterozygous variant in the domains of TACI. Among non-IEI genes, we identified other genes with a significantly higher mutation frequency in TACI patients compared with unsolved PAD patients (top 50 genes shown in Table S4). There was no enrichment associated with specific signaling pathways; however, some specific genes with important functions in B cell function and plasma cell development were significantly more often mutated in TACI patients, including *VEGFC* (P = 4.60E-08), *SEMA4D* (P = 2.29E-05), *BRD2* (P = 2.75E-05), *RASGRP3* (P = 2.48E-04), and *SOX1* (P = 4.10E-04).

**Figure 2.**
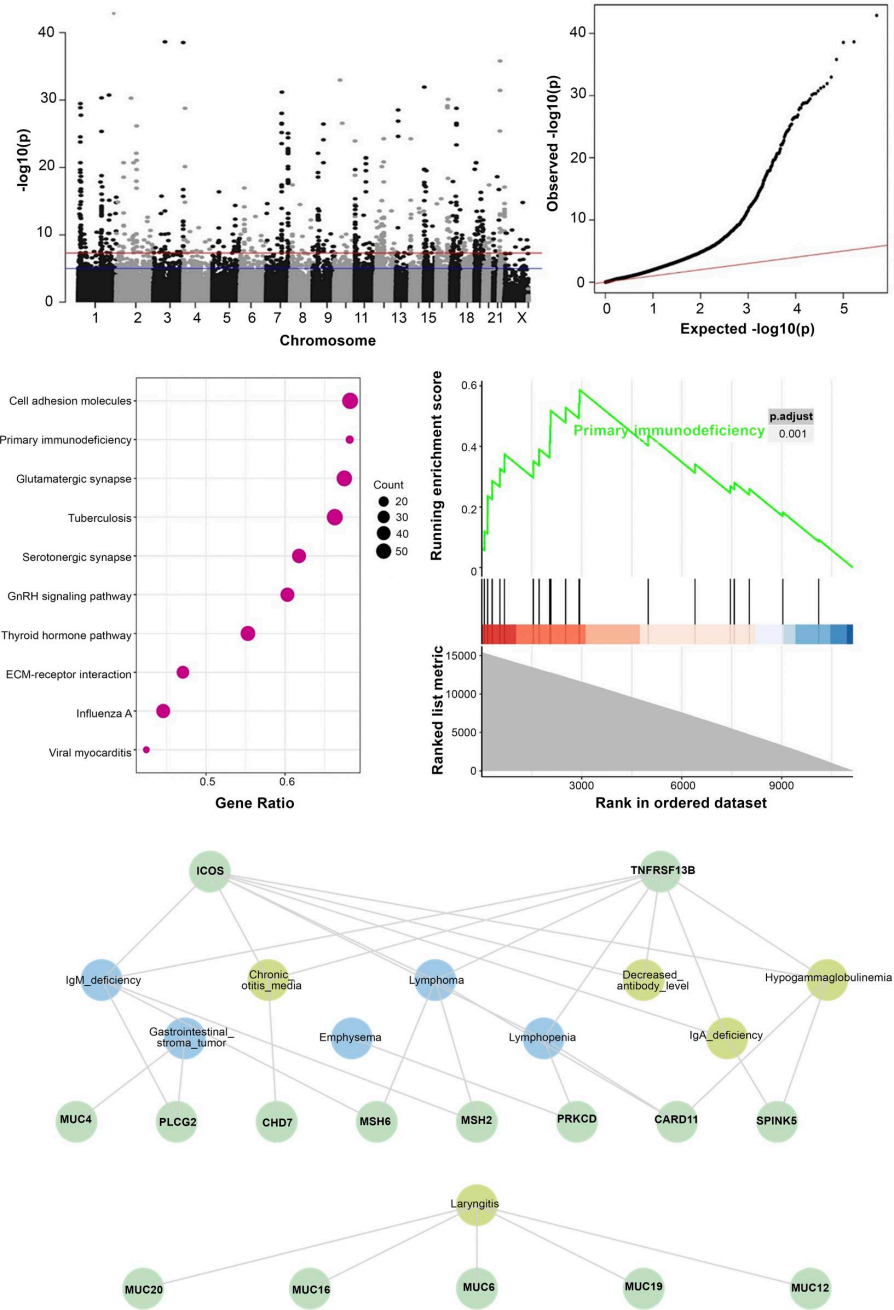
**GWAS analysis of modifier gene polymorphisms between 161 TACI patients and 1,241 unsolved PAD patients.** Enrichment analysis of significant modifier gene polymorphism using gene set enrichment analysis (GSEA), based on KEGG (yellow color) and HPO (blue color) ontology for significant SNPs identified in PAD patients with heterozygous *TNFRSF13B* variants.

Next, we tested for the presence or absence of polymorphic variants using the genome-wide association analyses (GWAS) between the TACI and unsolved PAD groups to see the impact on non-rare variants as well (Fig. 2). Intriguingly, the single nucleotide polymorphisms (SNPs) significantly enriched in these analyses are in or near genes known to be important for B cell development and antibody production (Fig. 2 and Table S5). Genetic polymorphisms in mismatch repair genes and genes of the PI3K pathway (e.g., *MSH2*, *MSH6*, *PRKCD*, and *PLCG2*) were more frequently observed in TACI patients (2–12 folds), mainly in patients with monoallelic *TNFRSF13B* mutations. Another interesting observation was the significantly lower presence of specific SNPs in the *TNFRSF13B* gene (e.g., rs2274892, P = 1.44E-13) among TACI patients (Table S5).

Since antigen presentation via human leukocyte antigen (HLA) regulates B cell activation, proliferation, and differentiation during cognate B cell–T cell interactions, we also typed and compared the HLA class I and II alleles between TACI patients and other unsolved PAD cohorts (Tables 4 and 5). Because of a previously reported association of HLA class I alleles with sIgAD or PAD (1, 41, 42, 43), it was surprising that all significant markers belonged to HLA class II. Among significantly associated class II markers, two were identified in the analysis; one significantly and exclusively expressed in TACI patients (DPA1*03, 27.9% vs. 0%, P = 3.75E-08) and another almost absent in TACI patients (DRB1*15, 8.0% vs. 51.0%, P = 7.41E-11). Of note, the observed association was independent of the ethnicity of patients (DPA1*03 observed in 45 TACI patients, originating from all three different PAD cohorts evaluated, Fig. S1). The HLA association was also independent of mutations in the genes *TAP1* and *TAP2*, which are non-HLA genes located near the HLA region on human chromosome 6 and encoding proteins involved in antigen presentation (Table S1, from seven patients with heterozygous TAP1/2 carriers none had DPA1*03). Other significantly enriched HLA markers in TACI patients were DPB1*55, DQA1*02, DRB1.04, and DRB1*03 (Table 5).

**Table 4.** Comparison of HLA class I markers between 161 TACI patients and 1,241 unsolved PAD patients

HLA	% in TACI patients	% in Other PADs	P value	FDR-adjusted P value	HLA	% in TACI patients	% in Other PADs	P value	FDR-adjusted P value
A*01	8.1	18.0	0.058447	0.409132	B*42	0.0	1.0	1	1.615385
A*02	18.0	18.0	0.87997	1.637412	B*44	1.9	14.0	0.004143	0.055364
A*03	11.8	12.0	1	1.814815	B*45	1.2	0.0	1	1.597826
A*11	8.1	16.0	0.127714	0.647377	B*46	0.0	1.0	1	1.580645
A*23	3.1	1.0	0.613505	1.346049	B*47	0.0	1.0	1	1.56383
A*24	16.8	7.0	0.050186	0.388283	B*49	3.7	1.0	0.36503	1.16651
A*25	1.2	2.0	1	1.792683	B*50	5.0	1.0	0.213669	0.848901
A*26	6.8	2.0	0.172446	0.745577	B*51	13.0	9.0	0.497786	1.143353
A*29	1.9	4.0	0.678498	1.366291	B*52	6.8	1.0	0.071197	0.418638
A*30	3.7	8.0	0.37173	1.092887	B*53	1.2	2.0	1	1.547368
A*31	1.9	2.0	1	1.771084	B*54	0.0	1.0	1	1.53125
A*32	8.1	5.0	0.5662	1.280482	B*55	3.7	4.0	1	1.515464
A*33	3.7	1.0	0.36503	1.219533	B*56	1.2	1.0	1	1.5
A*34	0.0	1.0	1	1.75	B*57	1.9	3.0	1	1.484848
A*66	1.2	1.0	1	1.729412	B*58	1.9	6.0	0.279016	0.976557
A*68	3.7	0.0	0.129714	0.635601	B*78	1.2	1.0	1	1.47
A*69	1.2	0.0	1	1.709302	C*01	3.7	4.0	1	1.455446
A*74	0.0	4.0	0.129714	0.615098	C*02	1.2	4.0	0.36503	1.14169
B*07	3.1	4.0	1	1.689655	C*03	1.9	8.0	0.104757	0.592283
B*08	3.7	14.0	0.026165	0.256421	C*04	24.2	12.0	0.042911	0.350438
B*13	5.0	2.0	0.441587	1.22478	C*05	1.2	6.0	0.123797	0.674005
B*14	3.7	1.0	0.36503	1.192432	C*06	9.9	9.0	1	1.441176
B*15	1.9	3.0	1	1.670455	C*07	19.9	20.0	1	1.427184
B*18	3.7	5.0	1	1.651685	C*08	3.1	2.0	1	1.413462
B*27	1.2	3.0	0.620596	1.303251	C*12	16.1	9.0	0.199543	0.838082
B*35	19.3	9.0	0.066645	0.408198	C*14	3.7	8.0	0.37173	1.071458
B*37	0.0	2.0	0.477289	1.252885	C*15	8.1	8.0	1	1.4
B*38	5.0	1.0	0.213669	0.872481	C*16	1.9	4.0	0.678498	1.347828
B*39	1.9	3.0	0.991011	1.820983	C*17	3.7	3.0	1	1.386792
B*40	3.1	6.0	0.495117	1.155273	C*18	0.0	1.0	1	1.373832
B*41	4.0	3.0	1	1.633333	​	​	​	​	​

**Table 5.** Comparison of HLA class II markers between 161 TACI patients and 1,241 unsolved PAD patients

HLA	% in TACI patients	% in Other PADs	P value	FDR-adjusted P value	HLA	% in TACI patients	% in Other PADs	P value	FDR-adjusted P value
DPA1*01	52.17	75.02	0.001232	0.02588	DPB1*33	1.24	0.00	1	1.105263
DPA1*02	18.01	24.98	0.301735	1.031514	DPB1*367	1.86	0.00	0.477289	1.150189
DPA1*03	**27.95**	**0.00**	**3.75E-08**	**2.76E-06**	DPB1*369	1.24	0.00	1	1.097015
DPA1*04	1.86	0.00	0.477289	1.230904	DPB1*37	1.24	0.00	1	1.088889
DPB1*01	1.24	2.01	1	1.361111	DPB1*39	1.86	0.00	0.477289	1.131638
DPB1*02	1.86	14.02	0.004143	0.05075	DPB1*400	1.24	0.00	1	1.080882
DPB1*03	4.97	2.01	0.441587	1.202099	DPB1*414	1.24	0.97	1	1.072993
DPB1*04	14.29	16.04	0.843022	1.588772	DPB1*416	1.86	0.97	1	1.065217
DPB1*05	1.24	2.98	0.613505	1.326254	DPB1*45	1.24	0.97	1	1.057554
DPB1*08	1.24	0.00	1	1.348624	DPB1*46	1.24	0.00	1	1.05
DPB1*09	1.24	2.01	1	1.336364	DPB1*463	0.00	14.02	0.000315	0.015427
DPB1*10	3.73	0.97	0.36503	1.117905	DPB1*49	0.00	0.97	1	1.042553
DPB1*104	1.24	2.01	1	1.324324	DPB1*50	1.24	0.00	1	1.035211
DPB1*105	3.11	2.01	1	1.3125	DPB1*55	**13.04**	**0.00**	**0.000578**	**0.01698**
DPB1*11	1.86	0.00	0.477289	1.209682	DPB1*59	3.11	0.00	0.244643	0.899064
DPB1*121	3.11	0.00	0.244643	0.922116	DPB1*648	0.00	7.01	0.020969	0.220172
DPB1*123	1.24	0.00	1	1.300885	DPB1*70	1.24	0.00	1	1.027972
DPB1*124	1.24	0.00	1	1.289474	DPB1*85	1.24	0.00	1	1.020833
DPB1*126	1.24	0.00	1	1.278261	DPB1*93	1.24	0.00	1	1.013793
DPB1*13	1.86	4.03	0.678498	1.329856	DQA1*01	24.22	27.96	0.628656	1.301584
DPB1*134	1.86	0.00	0.477289	1.189179	DQA1*02	**19.88**	**4.03**	**0.001099**	**0.026918**
DPB1*137	1.24	0.00	1	1.267241	DQA1*03	21.74	17.00	0.475297	1.270339
DPB1*14	1.24	2.01	1	1.25641	DQA1*04	21.74	19.02	0.726105	1.3862
DPB1*143	1.24	0.00	1	1.245763	DQA1*05	11.18	31.99	0.000577	0.021189
DPB1*15	1.86	2.01	1	1.235294	DQB1*02	24.84	48.03	0.001232	0.022645
DPB1*162	1.86	0.00	0.477289	1.169359	DQB1*03	29.81	26.03	0.636602	1.299728
DPB1*17	1.86	2.98	1	1.225	DQB1*04	6.21	0.97	0.123797	0.649933
DPB1*19	3.73	0.97	0.36503	1.095091	DQB1*05	21.12	14.02	0.264173	0.947159
DPB1*199	1.24	0.00	1	1.214876	DQB1*06	18.01	11.04	0.228222	0.88286
DPB1*20	1.24	0.00	1	1.204918	DRB1*01	11.18	2.98	0.052385	0.385027
DPB1*210	1.24	0.00	1	1.195122	DRB1*03	**13.04**	**0.97**	**0.0023**	**0.033807**
DPB1*219	1.24	0.00	1	1.185484	DRB1*04	**16.15**	**2.01**	**0.001318**	**0.021524**
DPB1*22	1.24	0.00	1	1.176	DRB1*07	4.97	7.98	0.5662	1.261081
DPB1*23	3.11	0.97	0.613505	1.307033	DRB1*08	6.21	0.00	0.038213	0.351078
DPB1*242	1.24	0.00	1	1.166667	DRB1*09	3.73	9.99	0.165843	0.738756
DPB1*26	1.24	0.00	1	1.15748	DRB1*10	1.86	0.97	1	1.006849
DPB1*260	0.00	0.97	1	1.148438	DRB1*11	6.21	14.99	0.064993	0.415389
DPB1*264	1.24	0.00	1	1.139535	DRB1*12	6.21	0.00	0.038213	0.330427
DPB1*28	3.73	9.99	0.165843	0.761842	DRB1*13	8.70	2.01	0.062749	0.419275
DPB1*30	1.24	0.00	1	1.130769	DRB1*14	8.07	4.03	0.37173	1.050853
DPB1*300	1.24	0.00	1	1.122137	DRB1*15	8.07	51.01	7.41E-11	1.09E-08
DPB1*31	0.00	7.98	0.01154	0.130491	DRB1*16	4.97	2.98	0.718216	1.389181
DPB1*316	1.24	0.00	1	1.113636	​	​	​	​	​

Red color depicts the significant preventive HLA markers. Bold indicates significant risk HLA markers.

## Discussion

Since the discovery of the association of PAD with genetic variants in *TNFRSF13B* (1, 14, 28), it has been noted that ∼10% of PAD patients carry these heterozygous variants, but their penetrance appears to be incomplete, as ∼1% of the healthy population also carry these variants (27). Although the causality of some frequent heterozygous mutations including p.C104R and p.A181E had been doubted, several functional assays have proven disruption of ligand binding and TM function for these heterozygous variants (4, 22, 37). Of note, ∼16% of TACI heterozygous carriers presenting with PAD in our study were found to also carry pathogenic variants in other known IEI genes with complete Mendelian inheritance. Most of these patients were tested using targeted sequencing gene panels and labeled as TACI diagnosis before conducting WES, as it was matched with their clinical and immunological profile. Therefore, our findings encourage clinical immunologists, who treat TACI-labeled PAD patients, to revisit the molecular diagnosis if the WES or whole genome sequencing is not performed.

Previous reports indicated a lower rate of autoimmune manifestations in biallelic cases compared with monoallelic ones (12), and one possible mechanistic explanation was provided (7). This phenotypic contrast is in line with our findings demonstrating a higher age of onset and longer diagnostic delay in the biallelic group. However, sinusitis and bronchiectasis were more common in patients with biallelic mutations as were lymphoproliferative complications. Our study indicates that TACI patients suffer from an increased risk of lower respiratory infections, autoimmunity, atopy, and malignancy, independent of the zygosity of the mutation in *TNFRSF13B* (hetero- or homozygous mutation carrier). Therefore, additional risk factors might contribute to the development of PAD (7, 44). One of these may be control of the expression of the different TACI isoforms, and the second is the location of the TACI mutation, as only the truncated version is associated with B cell maturation (35).

Earlier studies also raised the possibility that joint inheritance of *TNFRSF13B* mutations and HLA-associated susceptibility haplotypes might facilitate the development of immune deficiency and help explain variations in penetrance: Salzer et al. (1) reported that of 13 affected individuals, 9 had inherited HLA*B8 and 6 had inherited HLA*B44. Other studies also confirmed that the overall pattern of HLA types (HLA *DQ2, *DR7, *DR3-17, *B8, and/or *B44) in individuals with TACI deficiency seems to be different than in individuals with idiopathic CVID (43). Although previous studies mainly focused on HLA class I, our findings demonstrate class II variants in DPA1*03 (risk haplotype) and DRB1*15 (preventive haplotype). The association of specific class II alleles with the observed phenotype is conceivable because in secondary lymphoid organs, follicular B cells present peptides via HLA class II to receive CD4^+^ T cell help to produce specific antibodies (45). Co-stimulation by the CD40:CD40L interaction promotes cognate interaction with follicular helper T cells. This leads to B cell differentiation into centroblasts and centrocytes, which are organized in germinal centers with a B cell-rich dark zone. Centroblasts then undergo somatic hypermutation, producing clonal variations of germinal center B cells with improved antigen affinity and specificity (46, 47). Recent studies also indicated that HLA-DP polymorphisms may affect disease phenotypes and the antigenic peptide repertoire by altering interactions with the invariant chain. Specific HLA-DP polymorphisms block the association of IC antigens that have been degraded by the proteasome and taken up into the endoplasmic reticulum by the transporter associated with antigen processing (TAP), restricting both disease and self-associated antigens (48) from directly stimulating CD4^+^ T cells via HLA class II expressed on target cells (49). Specifically, the DPA1*03 has been linked recently with cutaneous drug adverse reaction (50) and molecular mimicry autoimmunity after *Salmonella typhi* infection (51), which both can be antibody mediated.

Recently, the transcriptome and proteome profiles of unstimulated and CD40L/IL21-stimulated naive B cells from affected individuals carrying the heterozygous C104R mutation were compared with those of unaffected carrier relatives who were in good health (27). According to this investigation, compared with healthy carriers, PAD patients with the *TNFRSF13B* mutation had 8% less accessible chromatin in unstimulated naive B cells and 25% less accessible chromatin in class-switched memory B cells. The ETS, IRF, and NF-kB transcription factors were represented by the most enriched transcription factors binding motifs in *TNFRSF13B*-mutant carriers. The NF-kB and MAPK pathway dysregulation was confirmed by validation tests. Naive B cells displayed elevated cell death pathways and decreased cell metabolism pathways in a steady state; however, following stimulation, there were increased immunological responses and decreased cell survival.

**Figure 3.**
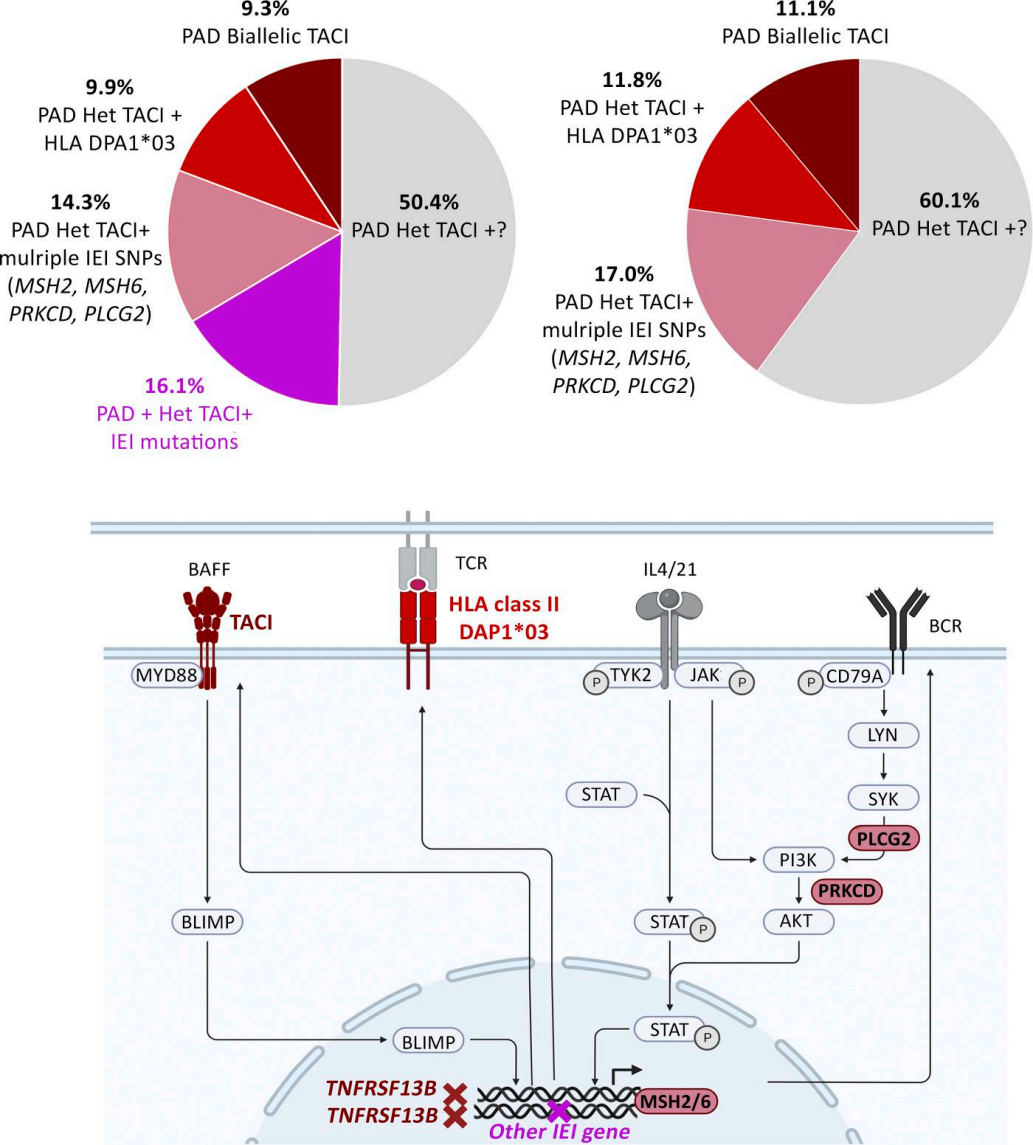
**Overview of the findings of the WES of patients with monoallelic and biallelic variants in *TNFRSF13B*.** The proportion of each etiology has been depicted with (left pie chart) or without (right pie chart) the inclusion of other known IEI pathogenic mutations with complete Mendelian inheritance.

A major limitation of our study design is that we collected data only from individuals affected by PAD. One alternative approach would be to collect WES data on unaffected *TNFRSF13B* mutation carriers/relatives, which would enable one to use family based statistical tests on putative genetic modifiers. Another approach would be to collect population-based WES data from unaffected carriers and to look for protective modifiers, but this would require a very large data set from the same original geographical region due to the fact that *TNFRSF13B* mutations are not common. By way of comparison, the advantage of our approach is that it maximizes the utility of modifiers gene identification in unsolved PAD cases compared with TACI-PAD cases. Investigating the valuable PAD resources from three different major cohorts worldwide, we re-examined all TACI variants in a patient-based setting and identified significant independent modifying factors influencing the penetrance of the antibody deficiency in at least 50% of the cohort (Fig. 3). In the remaining patients, the presence of unique HLA haplotypes, specific polymorphisms, or even unknown immune gene mutations are expected. The results of this study suggest a change in the paradigm of single gene analysis among clinical immunologists and indicate that several different digenic or even polygenic pathogenic hits (e.g., HLA, SNPs, and multiple IEI genes) may be responsible for the phenotype of antibody deficiency.

## Materials and methods

### Patients and clinical evaluation

A total of 161 symptomatic PAD patients with mutations in *TNFRSF13B* were recruited into this study between 2019 and 2023 for further genetic evaluation. These came from the Children’s Medical Center (Tehran, Iran, *n* = 64), the Center for Chronic Immunodeficiency (Freiburg, Germany, *n* = 47), and the Icahn School of Medicine at Mount Sinai (New York, USA, *n* = 50). Informed consent (including explanations about the risks and benefits of research-based next-generation sequencing) for the performed evaluations was obtained from all patients and/or their parents, according to the principles of the respective local ethics committees. An evaluation document was used to summarize the demographic information of the patients, including gender, year of birth, clinical parameters, previous medical history, family history, consanguinity of parents, and laboratory and molecular data. Complete blood count, lymphocyte subpopulations, B cell subsets, serum Ig levels, and specific antibody response were measured as previously described (52). Immunologic tests were repeated for each patient approximately every 6 mo during routine follow-up visits after the time of diagnosis to evaluate the progression of their antibody deficiency. All patients were diagnosed based on the updated clinical diagnostic criteria of the European Society for Immunodeficiencies (53). All patients were re-evaluated for fulfilling either the probable or possible diagnostic criteria, and secondary causes of PAD were excluded.

### Genetic analysis and diagnosis in unsolved patients

Genomic DNA was extracted from the whole blood of the patients and WES was performed using a pipeline described previously (31, 54). Annovar was used for mutation annotation, particularly for determining if a variant has previously been deposited in the Single Nucleotide Polymorphism Database (dbSNP) (55); dbSNP accepts pathogenic variants, so the presence of a variant in dbSNP with a low allele frequency does not imply that the variant is benign. Minor allele frequencies (MAFs) of variants in gnomAD (56) were recorded. Candidate variants were evaluated by the CADD algorithm, and an individual gene cutoff given by using the MSC was considered for impact predictions (57). The pathogenicity of all disease-attributable gene variants was re-evaluated using the updated guidelines for interpretation of molecular sequencing by the ACMG criteria (58, 59), considering zygosity/mode of inheritance, the allele frequency in the population, computational data (mainly for missense mutations using AlphaMissense software [60]), immunological data, and clinical phenotyping. All identified rare IEI variants (MAF < 0.01 based on gnomAD) in patients are identified for the first analysis. If detected, genetic changes in known IEI genes (10) with the expected Mendelian inheritance pattern and fulfilling the ACMG criteria were assigned as the main potential genetic cause; other pathogenic/likely pathogenic IEI gene rare variants with a pattern of incomplete Mendelian inheritance not consistent with IUIS classification of that gene or other nonpathogenic rare variants with complete Mendelian inheritance were referred to as potential modifiers. PAD patients with unsolved genetic diagnosis after WES analysis were used as the control for IEI modifier (damaging mutation but in non-Mendelian inheritance) and genome-wide association studies (polymorphism variants with MAF ≥ 0.01). Mutations observed in *TNFRSF13B* were partly classified based on the three domains of the encoded TACI protein (UniPort accession no. O14836): EC (positions 1–165), TM (positions 166–186), and IC (positions 187–293) (61).

### Statistical analysis

Different parameters between patients’ groups were compared. A one-sample Kolmogorov–Smirnov test was applied to estimate whether the data distribution was normal. Parametric and nonparametric analyses were performed based on the findings of this evaluation. Statistical analysis was performed using SPSS (version 21.0.0, SPSS) and R statistical systems (version 3.4.1., R Foundation for Statistical Computing). Several genetic models have been tested, including zygosity of TACI variants(s), affected TACI domain, TACI mutation type, the role of other IEI mutations, the presence of other IEI modifiers, and the impacts of other non-IEI genes (e.g., novel mutations, HLA typing, and GWAS). The false discovery rate (FDR) was used as a statistical approach in multiple hypothesis testing to correct for multiple comparisons in IEI modifier and GWAS using PLINK (62). For GWAS analysis, we only considered the polymorphic variants annotated by dbSNP version 138 as located on an autosome or on the X chromosomes with MAF ≥0.01 and imputation quality Rsq ≥ 0.3. Enrichment analysis of pathways and genes was performed using the gene set enrichment analysis package in version 2.6.0 of Bioconductor. Proteins with significant changes in abundance were further analyzed using the Enrichr Gene Ontology Biological Process 2021 term enrichment (63). The t-statistic mean of the possibly enriched gene sets was computed based on the human phenotype ontology pathway (64). Using a permutation test with 1,000 repetitions, the cutoff of significance level P value was chosen as 0.05 for the significant pathways associated with mutations in *TNFRSF13B*. HLAminer was used to detect the HLA class I and HLA class II alleles in each patient from the WES reads as previously described (65). Chi-square tests with FDR correction were utilized to compare individual HLA types between the 161 TACI group and 1,241 control PAD group, where we counted the number of each allele with four digits of precision regardless of whether the alleles were in the heterozygous state or the homozygous state.

### Online supplemental material

The supplementary information includes one figure and five tables. Fig. S1 visualizes the frequencies of the most common *TNFRSF13B* variants and significantly associated HLA alleles in PAD patients from three different cohorts. Table S1 and S2 provide a comprehensive list of rare exonic variants in IEI genes detected in the WES of the 161 PAD study subjects with variants in *TNFRSF13B*. Table S3 shows the top 50 known IEI genes with rare modifier variants whose frequencies are most statistically significantly different between 161 PAD study subjects with variants in *TNFRSF13B* and 1,241 control PAD study subjects that do not have variants in *TNFRSF13B*. Table S4 is structurally identical to Table S3 but instead includes the top 50 genes with rare variants in non-IEI genes that distinguish the 161 cases from the 1,241 controls. Table S5 is structurally similar but instead includes known non-rare polymorphisms with dbSNP identifiers that have different frequencies in the 161 cases compared with the 1,241 controls.

## Supplementary Material

Table S1 provides a comprehensive list of rare exonic variants in IEI genes detected in the WES of the 161 PAD study subjects with variants in *TNFRSF13B*.

Table S2 provides a comprehensive list of rare exonic variants in IEI genes detected in the WES of the 161 PAD study subjects with variants in *TNFRSF13B*.

Table S3 shows the top 50 different IEI modifier gene variants (all rare variants) between 161 TACI patients and 1,241 unsolved PAD patients.

Table S4 shows the top 50 different non-IEI modifier gene mutations (all rare variants) between 161 TACI patients and 1,241 unsolved PAD patients.

Table S5 shows the main modifier gene polymorphism in the significantly enriched pathways between 161 TACI patients and 1,241 unsolved PAD patients.

## Data Availability

The raw data supporting the conclusions of this article will be made available by the authors, without undue restrictions. Because the data include germline mutations of human subjects, the data cannot be made publicly available without restrictions so as to protect the privacy of the study subjects.
