# Supplementary material for: Re-evaluation of the contribution of TNFRSF13B variants to antibody deficiency
Source: J Hum Immun. 2025 Aug 19;1(4):e20250016. doi: 10.70962/jhi.20250016 (PMC12435966; doi:10.70962/jhi.20250016)
Supplement: Table S2 — provides a comprehensive list of rare exonic variants in IEI genes detected in the WES of the 161 PAD study subjects with variants in TNFRSF13B. [file jhi_20250016_tables2.docx]

| **Table S2 –** List of rare non-synonymous exonic variants in IEI genes in 161 PAD patients evaluated in this study. The red color depicts the pathogenic and likely pathogenic variants based on ACMG criteria which is matched with the complete Mendelian inheritance based on IUIS classification. Black color depicts non-pathogenic variants based on ACMG criteria but is matched with the complete Mendelian inheritance. The last column indicates the number of remaining non-synonymous exonic variants in the Table S1 file. | | | | | | | |
| --- | --- | --- | --- | --- | --- | --- | --- |
| **ID** | **TACI variants** | **TACI**  **Monoallelic/ Biallelic** | **Genetic defect in B cells** | **Genetic defect in T cells** | **Bone marrow failure defects** | **Other IEI genetic defects** | **Number of other**  **possibly IEI modifiers** |
| P1 | p.Cys104Arg | Het | - | - | TERT (hom p.Val28Ala) | - | 11 |
| P2 | p.Cys104Arg | Het | - | KMT2D (het p.Pro2210Leu) | SAMD9L (het p.Arg724His) | TBK1 (het p.Arg724His) | 8 |
| P3 | p.Cys104Arg | Het | NFKB2 (het p.Tyr5Cys) | AIRE (comp het p.Gly467Arg/p.Asp503Asn) | - | POLR3C (het p.Ser512ValfsTer20) | 8 |
| P4 | p.Cys104Arg  p.Leu69ThrfsTer12 | Comp Het | - | STAT1 (het p.Glu6Lys) | - | MEFV (het p.Gly437TrpfsTer20)  CXCR4 (het p.L47F)  SH3BP2 (het p.His242ThrfsTer36) | 6 |
| P5 | p.Ala181Glu  p.Cys104Arg | Comp Het |  |  | SAMD9 (het p.Ser627PhefsTer16) | NLRP2 (het p.Ile1039Val) | 6 |
| P6 | p.Cys104Arg | Het | - | - | - | - | 16 |
| P7 | p.Cys104Arg | Het | - | - | - | - | 6 |
| P8 | p.Cys104Arg | Het | TNFRSF13C (comp het p.His159Tyr/ p.Pro21Arg) | - | - | HCK (het p.I127N)  APOL1 (het p.Leu174Phe) | 9 |
| P9 | p.Cys104Arg | Het | - | - | - | NLRP1 (het p.Gly106Arg)  NLRP 2(het p.Thr403Arg/)  NBAS (comp het p.Arg1073Cys / p.Tyr1023His)  RANBP2 (het p.Thr2602Met) | 9 |
| P10 | p.Cys104Arg | Het | - | - | - | NLRP2 (het p.Thr403Arg) | 11 |
| P11 | p.Cys104Arg | Het | IRF2BP2 (het p.Ala107Thr)  NFKB1 (het p.His712Gln) | RELA (het p.Leu286Val)  KMT2A (het p.Met2175Val) | DKC1 (hom p.Ser280Arg) | POLA1 (hom p.Lys750Arg)  STXBP2 (het p.Lys208Gln)  NLRP1 (het p.Val1329Ile)  NOD2 (het p.Ala755Val)  PSTPIP1 (he p.Ala346Val) | 9 |
| P12 | p.Cys104Arg | Het | - | TGFBR2 (het p.Ser553Thr) | TERT (het p.Gly32Arg) | - | 5 |
| P13 | p.Cys104Arg | Het | - | RAG2 (hom p.Lys498Ter) | - | NLRP2 (het p.Thr403Arg) | 10 |
| P14 | p.Cys104Arg | Het | - | RELA (hetp.Arg168Ser) | - | STXBP2 (het p.Arg529Pro) | 3 |
| P15 | p.Ile87Asn | Het | - | TET2 (comp het p.His248Gln/p.Gly429Arg) | - | RANBP2(hetp.Leu361Phe)  SYK(hetp.Met450Ile) | 10 |
| P16 | p.Arg72His | Het | - | CTLA4 (het p.F179Cfs*28)  TGFBR2 (het p.Lys128SerfsTer35) | - | TICAM1 (het p.Ser160Phe)  MECOM (het p.Arg780Lys) | 5 |
| P17 | p.Arg72His | Het | TCF3 (het p.His269Arg) | FAT4 (comp het p.Asn1309Ser/ p.Ile697Thr) | - | NLRP3 (het p.Arg675Trp) | 5 |
| P18 | p.Cys172Tyr | Het | - | - | - | TLR8 (hom p.Thr538Lys)  NLRP1 (het p.Gly106Arg)  NCSTN (het p.Asn417Tyr) | 10 |
| P19 | p.Cys193Arg  p.Ser144Ter | Comp Het | - | KMT2D (het p.Met3398Val)  TGFBR2 (het p.Lys128SerfsTer35) | - | CFHR5(het p.Glu163LysfsTer10)  IRF4(het p.Arg8Gln) | 8 |
| P20 | p.Phe21SerfsTer2 | Het | TCF3 (het p.Gly385Asp) | - | - | NLRP2(het p.Pro576Leu)  CFH(het p.Gln950His)  GFI1(het p.Pro107Ala)  ATAD3A(het p.Val86Met) | 6 |
| P21 | p.Arg72His | Het | - | KMT2D (het p.Pro998Thr) | - | POLR3A (het p.Ile980Val)  PLCG2 (hetp.Pro522Arg)  CARD14 (hetp.Arg610Cys) | 3 |
| P22 | p.Pro97Arg | Het | NFKB1 (het p.Ala245Val) | KMT2A (het p.Glu502Lys  AIRE (het p.Val199Ile | - | ELF4 (hom p.Thr187Asn)  NLRP3 (het p.Ser161Ile)  KRAS (het p.K180del)  C3 (het p.Leu9Pro)  NLRP2 (het p.Ser626Pro) | 7 |
| P23 | p.Arg202His | Het | - | CTLA4 (het p.Gly109Glu)  KMT2D (het p.Arg2401His) | - | PLCG2 (het p.Leu835Ile)  SERPING1 (het p.Thr95Pro)  TNFRSF1A (het p.Asp41His)  ELANE (het p.Asn124His)  CXCR4 (het p.L47F) | 8 |
| P24 | p.Arg202His | Het | - | CHD7 (het p.Met340Val) | - | - | 5 |
| P25 | p.Ile87Asn | Het | - | - | - | RANBP2 (het p.Ser1981Thr) | 11 |
| P26 | p.Leu171Arg | Het | TCF3 (het p.R556W) | TTC7A (comp hetp.Lys252Arg/ p.Ser318Pro) | RTEL1 (het p.V1179M) | GATA2 (het p.Gly100Trp)  C1S(het p.Asp315Asn) | 10 |
| P27 | p.Phe185Cys  p.Leu69ThrfsTer12 | Comp Het | TCF3 (het p.Gly385Asp) | KMT2D (het p.Gly2671Ser) | - | MEFV (het p.Arg354Trp)  ATAD3A (het p.Val160Met)  CFH (het p.Gln1076Glu) | 10 |
| P28 | p.Phe185Cys  p.Leu69ThrfsTer12 | Comp Het | - | ITPKB (comp het p.Val914Ile/p.Glu62Lys) | - | NLRC4 (het p.Glu196LysfsTer11)  CFH (het p.Gln1076Glu) | 6 |
| P29 | p.Cys104Arg | Het | - | BACH2 (het p.Ala195Thr) | SAMD9(het p.Gly1575Glu) | VPS13B (comp het p.Thr1271Ser/ p.Ser2571Phe)  PLCG2 (het p.Lys1248Gln)  NLRP1(het p.Gln533His)  RANBP2 (het p.Ser1981Thr)  HCK (het p.T270M) | 7 |
| P30 | p.Cys104Arg  p.Cys104Arg | Hom | - | - | - | VPS13B (comp het p.Thr1271Ser/ p.Ser2571Phe)  CARD14 (het p.R941Q)  RANBP2 (het p.Ser1981Thr) | 7 |
| P31 | p.Gln57His | Het | - | - | - | - | 10 |
| P32 | p.Ile87Asn | Het | - | TGFBR2 (het p.Ser553Thr) | RTEL1 (het p.T144I) | RANBP2 (het p.Thr2391Ser) | 5 |
| P33 | p.Ile87Asn | Het | - | TGFBR2 (het p.Ser553Thr) | RTEL1 (het p.T144I) | NLRC4(het p.D1011del  RANBP2 (het p.Thr2391Ser) | 6 |
| P34 | p.Ala181Glu | Het | - | CHD7(het p.Tyr1075Cys) | - | NLRP2(het p.Phe356Leu) | 8 |
| P35 | p.Ala181Glu | Het | - | MCM10 (comp het p.Lys480Asn/ p.Thr741Lys) | - | NLRP2 (het p.Phe356Leu) | 14 |
| P36 | p.Ala181Glu  p.Cys104Arg | Comp Het | - | - | - | UNC93B1 (hom p.Gly592Glu)  TLR3 (het p.Ile760Val)  CFH (het p.Gln950His)  NOD2 (het p.Met863Val) | 11 |
| P37 | p.Ala181Glu | Het | - | - | SAMD9 (het p.Glu1368Lys) | - | 7 |
| P38 | p.Cys104Arg | Het | NFKB1 (het p.Arg214Gln) | - | RTEL1(het p.Met320Thr) | CFHR3 (het p.Pro241Ser)  IRF4 (het p.Ala341Val) | 9 |
| P39 | p.Ala181Glu | Het | - | STAT3 (het p.Ser69Arg) | - | RANBP2 (het p.Thr2391Ser)  CASP10 (het p.Ile406Leu) | 10 |
| P40 | p.Ala181Glu | Het | - | TGFBR2 (het p.R49K) | RAD51(hom p.S104T) | CFHR4(het p.Gln142Leu)  NLRP1(het p.Ser679Asn)  CARD14(het p.R826W) | 8 |
| P41 | p.Cys104Arg | Het | TCF3 (het p.Arg438Gln) | PRKDC (comp het p.Leu1531Phe/ p.Ile1413Val)  BCL11B (het p.Thr335Met)  TGFBR2 (het p.Ala217Pro)  IKBKB (het p.R32G) | - | PSTPIP1(het p.Gln218His)  NOD2 (het p.Ala755Val)  RANBP2(het p.His777Arg)  HCK(het p.A349T) | 17 |
| P42 | p.Cys104Arg | Het | TNFSF12 (hetp.Ala106Gly) | IKBKB (het p.Q355H) | RTEL1(het p.S1193P) | - | 6 |
| P43 | p.Cys104Arg | Het | - | FOXN1(het p.Glu330Lys) | - | CYBB (hom p.Asp517Glu) | 12 |
| P44 | p.Cys104Arg | Het | - | RELA (het p.Arg168Ser) | - | - | 10 |
| P45 | p.Cys104Arg | Het | - | RELA (het p.Arg168Ser) | - | STXBP2 (het p.Arg529Pro) | 13 |
| P46 | p.Cys104Arg | Het | - | - | - | STXBP2 (het p.Arg529Pro) | 8 |
| P47 | p.Ala181Glu | Het | - | CHD7 (het p.Ala2209Thr)  RELA (het p.Asp288Asn) | - | - | 11 |
| P48 | p.Ala181Glu | Het | - | - | - | - | 8 |
| P49 | p.Ala181Glu | Het | - | IKZF2(het p.Pro341His)  KMT2D(hetp.Ile1696Val) | SAMD9 (het p.Thr205Pro | OAS1(het p.Asn307Ile)  PLCG2(het p.Thr26Met)  NLRP2(het p.Leu59Phe)  NLRC4(het p.D1011E) | 6 |
| P50 | p.Ala181Glu | Het | - | - | - | PLCG2(het p.Thr26Met)  NLRP2(het p.Leu59Phe)  NLRC4(het p.D1011E) | 8 |
| P51 | p.Ser194Tyr  p.Cys104Arg | Comp Het | - | - | SRP72(het p.Glu582Val)  BRCA2(comp het p.Gly1529Arg/ p.Gly1771Asp) | NFAT5 (het p.P31Q)  TNFAIP3 (het p.Arg697Lys) | 9 |
| P52 | p.Cys104Arg | Het | - | - | - | PSTPIP1 (hetp.Gln218His  NOD2(het p.Asn637SerfsTer120  TNFAIP3(het p.Arg697Lys) | 8 |
| P53 | p.Ser194Tyr  p.Cys104Arg  p.Pro35Leu | Comp Het | - | - | - | ACTB (het p.Val10Ile) | 7 |
| P54 | p.Cys104Arg | Het | TCF3 (het p.Gln390Pro) | STAT3 (het p.Pro715AsnfsTer8)  LIG1 (comp het p.Arg641Leu/ p.Thr415MetfsTer10) |  | RANBP2 (het p.Thr2656Ala)  IFIH1 (p.Leu554His)  TOP2B (p.Ser210Asn)  POLA1 (p.Lys750Arg) | 7 |
| P55 | p.Ser13Gly | Het | PIK3CD (het p.Glu1045Lys) | - | - | C1S (het p.Gly360Val)  NOD2 (het p.Pro668Leu)  RANBP2 (het p.Ser730Pro) | 12 |
| P56 | p.Arg67LysfsTer23 | Het | - | SEMA3E (het p.Asn595Asp) | - | POLR3C(het p.Ser512ValfsTer20)  TICAM1(het p.Ser186Leu)  NLRP2(het p.Thr403Arg)  TNFAIP3(het p.Ala545Val) | 9 |
| P57 | p.Cys104Arg | Het | - | FAS (het p.D317G)  MAN2B2 (comp het p.Val414Ile/ p.Gln788Arg) | - | OAS1(het p.G352S) | 12 |
| P58 | p.Leu69ThrfsTer12 | Het | - | AIRE(het p.Pro400Leu) | TP53(het c.-28-1G>T)  RTEL1(hetp.Gly340Ser) | PLCG2(hetp.Pro522Arg)  NLRP1(hetp.Gln533His)  SH3BP2(hetp.Ser427del) | 6 |
| P59 | p.Leu69ThrfsTer12 | Het | - | IKZF2(het c.158-4A>G) | - | PLEKHM1 (hom p.Glu98Lys)  CFHR5 (hetp.Met514Arg)  TRAF3 (hetp.Arg296Gln)  NLRP1(hetp.Gln533His)  IL17RA (comp hetp.Glu226Gln/ p.Glu699Gly) | 8 |
| P60 | p.Cys104Arg | Het | - | STAT5B (het p.Ser554Tyr) | - | IFIH1(het p.Glu627Ter) | 10 |
| P61 | p.Cys104Arg | Het | - | DOCK8 (comp het p.Arg475Gln/ p.Glu571Asp)  KMT2D(het p.Pro2271Ser) | - | SERPING1(het p.Asp284Asn) | 8 |
| P62 | p.Ile87Asn | Het | - | - | RTEL1(hetp.S1193P) | - | 7 |
| P63 | p.Cys104Arg | Het | - | DOCK8 (comp het p.Arg475Gln/ p.Lys21Arg)  AIRE(het p.Arg471Cys)  KMT2D(hetp.Val4305Ile) | - | RANBP2(hetp.Pro1055Ser | 5 |
| P64 | p.Cys104Arg  p.Leu69ThrfsTer12 | Comp Het | TCF3 (het p.Ala8Ser) | - | - | TLR3(hetp.Ser115LeufsTer11)  KRAS(het p.Tyr166Asn)  PLCG2(hetp.Pro1115Ser) | 7 |
| P65 | p.Tyr164Ter  p.Cys104Arg | Comp Het | - | - | - | ATAD3A (het p.Q142X)  SH3BP2 (het p.R39Sfs*30) | 5 |
| P66 | p.Leu171Arg | Het | - | KMT2D (het p.Pro2146Leu) | - | TMEM173 (comp het p.Val194Leu/ p.Leu126Ile)  POLR3C (het p.Gln371Glu) | 11 |
| P67 | p.Leu69ThrfsTer12 | Het | - | KMT2A (het p.Leu3576Pro) | - | - | 8 |
| P68 | p.Lys188Met | Het | - | IKZF3 (het p.Phe108Cys) | SLX4 (comp het p.Arg1372Gln/ p.Ala916Ser) | NOD2 (het p.Arg703His)  PLCG2 (het p.Leu848Phe)  POLR3C (het p.Gln371Glu) | 20 |
| P69 | p.Leu69ThrfsTer12 | Het | - | FAT4(comp het p.Ser736Arg/ p.Arg2285Trp)  IL6ST (het p.Pro434Arg) | - | APOL1(het p.Ala256Val) | 8 |
| P70 | p.Cys104Arg  p.Cys104Arg | Hom | - | KMT2D(het p.Glu913Lys) | RTEL1 (het p.G1218D) | APOL1(het p.Gly247Arg) | 10 |
| P71 | p.Leu69ThrfsTer12 | Het | TCF3(het p.I562V) | IKZF2(het p.Ser358Arg)  AIRE(het p.Gly424Asp) | MECOM(het p.Gln171Arg) | PLCG2(het p.Pro1115Ser) | 6 |
| P72 | p.Cys104Arg | Het | TCF3(het p.Ala8Ser) | - | - | TLR3(het p.Ser115LeufsTer11)  KRAS(het p.Tyr166Asn) | 9 |
| P73 | p.Cys104Arg | Het | - | - | - | - | 7 |
| P74 | p.Leu69ThrfsTer12 | Het | - | FAT4(comp het p.Ser736Arg/ p.Arg2285Trp) | - | TLR8(hom p.Met1033Thr)  GFI1(het p.Pro107Ala) | 13 |
| P75 | p.Cys104Arg | Het | NFKB2(het p.His98Asn) | - | - | MEFV(het p.Lys695Arg) | 11 |
| P76 | p.Lys188Met | Het | - | CHD7(comp het p.Gly744Ser/ p.Ala2160Thr)  AIRE(het p.Pro166Leu) | ACD (het p.Gly391Glu) | LYST(het p.Arg2624Trp/ p.Val1821Phe)  CARD14(het p.Arg597Trp)  ATAD3A(het p.G126D)  TICAM1(het p.Ser186Leu)  NLRP2(het p.Glu1029Val)  TNFRSF1A (het p.Pro75Leu) | 22 |
| P77 | p.Cys104Arg | Het | PTEN (het c.802-2A>T) | - | - | TBK1 (het c.1960-3C>T) | 8 |
| P78 | p.Ala181Glu | Het | - | IKBKB(het c.-18-4A>G) | - | CFHR5(het p.Glu146Ter)  C3(het p.Leu1318Arg)  SRP72(het p.Ile530Thr ) | 5 |
| P79 | p.Cys104Arg | Het | - | NFE2L2 (het p.Ile151Thr) | - | - | 10 |
| P80 | p.Cys104Arg  p.Cys104Arg | Hom | - | TGFBR1(het p.Ala26del) | - | CFHR4(het p.W258_E577del)  NLRP3(het p.Gly921Asp)  SERPING1(het p.Ala2Val)  MEFV(het p.Ile259Val)  NLRP12(het p.Gly448Ala)  TNFAIP3(het p.Arg572Gln) | 14 |
| P81 | p.Cys104Arg | Het | NFKB1 (het.Arg579Lys) | TGFBR2(het p.Tyr259Ser) | - | - | 10 |
| P82 | p.Ala181Glu | Het | - | TGFBR2 (het p.Thr206Met) | - | TBK1(het p.Val152Leu)  NLRP3(het p.Arg137His)  NLRP2(het p.Gln764Arg) | 15 |
| P83 | p.Cys104Arg | Het | - | FAS (het p.G247S) | - | NOD2(het p.Arg684Gln)  PLCG2(het p.Pro522Arg)  SYK(het p.Arg625Gln) | 9 |
| P84 | p.Arg202Cys | Het | - | - | RELA (het p.Asp288Asn) | EFL1(comp het p.Val1010Ile/ p.Pro881Thr)  PLCG2(het p.Leu848Phe)  C3(het p.Ser1619Arg)  APOL1(het p.Val369Glu) | 10 |
| P85 | p.Arg72His | Het | - | - | - | POLR3C(het p.Arg367His)  NLRP2(het p.Ala228Ser) | 6 |
| P86 | p.Cys104Arg | Het | - | IKZF2 (het c.158-4A>G) |  | POLR3A (hom p.Ile980Val) | 12 |
| P87 | p.Cys172Tyr | Het | - | RELA (het p.Asp288Asn) | ACD (het p.Ser421Asn) | NLRP12(het p.His304Tyr)  RANBP2(het p.Pro1055Ser) | 6 |
| P88 | p.Cys104Arg | Het | - | IKZF2 (het c.158-4A>G) | - | POLR3A(hom p.Ile980Val) | 9 |
| P89 | p.Ala181Glu | Het | - | - | - | EFL1(comp het p.Val1010Ile/ p.Pro881Thr)  TRAF3 (het p.Arg118Trp) | 8 |
| P90 | p.Cys104Arg  p.Ile87Asn | Comp Het | - | - | - | - | 5 |
| P91 | p.Cys104Arg | Het | - | - | - | CFTR (comp het p.Arg258Gly/ p.Leu454del)  RIPK1 (het p.Glu311Lys)  NOD2 (het p.Pro537Ser) | 8 |
| P92 | p.Cys104Arg  p.Cys104Arg | Hom | PIK3CD (het p.Val20Ile) | - | - | - | 14 |
| P93 | p.Cys104Arg | Het | TCF3 (het p.Ser295del) | - | - | NLRP12(het p.Ser934LeufsTer15)  ATAD3A(het p.Gly18Trp)  JAK1(het p.Leu922Met)  CFHR4(het p.Gln282Lys)  RANBP2(het p.Asp234Glu)  CXCR4(het p.V39L) | 13 |
| P94 | p.Cys104Arg | Het | TCF3(het c.550-4G>T) | FAS (het p.S333N) | FANCB (hom p.Thr210Ala)  SAMD9L (het p.Ala566Thr | KMT2D(het p.Ser4297Pro )  TTC7A(comp het p.Lys252Arg/p.Ser318Pro)  CXCR4(hetp.P55T) | 7 |
| P95 | p.Lys188Met | Het | - | BACH2(het p.Glu797Ter)  KMT2A(het p.Gly1053Ser)  IKZF2(het p.Met233Ile)  IKZF1(het p.Asn392His) | BRCA2(comp het p.Lys2339Asn/ p.His2440Arg)  FANCM(comp het p.Gly1014Cys/ p.Glu1521Asp)  SLX4(comp het p.Arg1372Gln/ p.Ala916Ser) | ATAD3A (het p.P19L)  TNFRSF1A (het p.Pro75Leu)  NOD2(het c.541-3T>C)  C3(het p.Leu1549Met)  RANBP2(het p.His777Arg)  THBD (het p.Gly502Arg)  TLR3(het p.Ser737Thr) | 33 |
| P96 | p.Arg72His | Het | - | MALT1(hom p.Lys128Asn) | - | POLR3C(het p.Arg367His)  SERPING1(het p.Thr95Pro)  CARD14(hetp.L942V)  NLRP2(het p.Ala228Ser) | 4 |
| P97 | p.Cys172Tyr | Het | - | RELA(het p.Asp288Asn) | ACD(het p.Ser421Asn) | NLRP12(het p.His304Tyr)  RANBP2(hetp.Pro1055Ser) | 4 |
| P98 | p.Gln57His | Het | - | - | - | IFNGR1 (hom p.I249Ffs*10)  CFHR4 (het p.G256R)  MEFV (het p.Gly304Arg) | 8 |
| P99 | p.Pro42Thr | Het | - | RAG1(hom p.Cys358Tyr)  IKZF2(hetp.Asn30Ser) | RTEL1(het p.D1261E) | XIAP (hom p.Ile389Val)  NLRP12(het p.Arg1030Gly)  POLR3A(het p.Pro918Ser) | 13 |
| P100 | p.Cys104Arg | Het | - | KMT2D (het p.Gln3919del)  CCBE1(comp het p.H186R/ p.P87Q)  IKBKB (het c.106-3T>C) | - | RANBP2(het p.Asn2068Ser)  POLR3F(het p.Ser208Gly)  IFNGR1(het p.Gln45Pro) | 10 |
| P101 | p.Cys104Arg | Het | - | KMT2D (het p.Pro647Gln) | - | - | 10 |
| P102 | p.Pro151Leu | Het | - | - | RTEL1(comp het p.Gly340Ser/p.Glu771del) | MAP1LC3B2 (het p.Met88Thr)  CASP10(het p.Ser216Phe) | 9 |
| P103 | p.Arg84Thr | Het | - | - | - | GATA2 (hetp.Pro250Ala) | 12 |
| P104 | p.Cys104Arg | Het | SPI1(het p.Arg139Gln) | - | - | TMC6(comp het p.Ser195Gly/ p.Glu142Lys)  NFAT5(het p.Gln812del) | 14 |
| P105 | p.Arg72Cys | Het | TCF3(het p.Ser295del) | - | - | IL6ST (hetp.Arg644Gln)  NLRP3(hetp.Thr954Met)  CFHR3(het p.Arg39His)  NFAT5(het p.Glu30Asp) | 9 |
| P106 | p.Pro42Thr | Het | - | - | SLX4 (het p.Pro1677Ser) | TMEM173 (hom p.Met271Val)  MEFV (het p.Arg461Gln)  TICAM1(het p.Val80Met)  NLRP12(het p.Arg1030Gly) | 17 |
| P107 | p.Phe21Leu | Het | TCF3(het p.Pro137Ser) | - | - | ATAD3A (het p.Trp230Arg)  C1R(het p.Gln380His)  NLRP12(het p.Arg352Cys)  IFIH1(het p.Thr702Ile)  SRP72(het p.Asn290Ser) | 8 |
| P108 | p.Arg122Trp | Het | - | NHEJ1 (hom ivs2 -1 C>A)  IKZF1 (het p.A138V) | - | - | 13 |
| P109 | p.Leu69ThrfsTer12 | Het | NFKB1 (het p.Leu587CysfsTer5) | NFE2L2(hetp.Ile157Thr)  TTC37(comp het p.Ala1450Ser/ p.Lys1354Arg) | RTEL1(het p.Ser815Ile) | - | 8 |
| P110 | p.Cys104Arg | Het | - | CFH(het p.Arg796Lys) | - | SOCS1(hom p.Glu142Gln)  CASP10(het p.Val18Met) | 8 |
| P111 | p.Arg14Cys | Het | - | - | DKC1 (hom p.Ser280Arg) | STXBP2(het p.Ser152Gly) | 8 |
| P112 | p.Ala181Glu | Het | - | BCL11B(het p.Ala544Val)  ERBB2IP(het p.Ile958Val) | - | - | 7 |
| P113 | p.Cys104Arg | Het | - | MAGT1 (hom p.Met46Val)  AIRE (het p.Arg471Cys) | - | TRAF3 (het p.Ser447Leu) | 10 |
| P114 | p.Cys172Tyr | Het | NFKB1 (het p.His712Gln) | CD27 (hom p.C96Y) | - | ATAD3A(het p.His551Tyr)  VPS13B ( hom p.Asn2808Thr) | 7 |
| P115 | p.Thr247Met | Het | - | AK2 (hom p.Ala182Asp) | - | - | 6 |
| P116 | p.Arg122Trp | Het | SPI1(het p.Gly157Arg) | KMT2D (het p.Arg1918Cys) | BRCA1(comp hetp.N550H/ p.Y179C) | EFL1(comp het p.Val1010Ile /p.Pro881Thr) | 9 |
| P117 | p.Cys104Arg | Het | NFKB1 (het p.Gly386Arg) | - | RTEL1(het p.S1141T) | CFHR3(het p.Cys175Tyr)  MTHFD1(het p.Gly301Ala)  MEFV(het p.Thr267Ile) | 5 |
| P118 | p.Cys104Arg | Het | - | ATM (hom p.Ala1299ProfsTer50)  CARD11(het c.683-4G>A) | - | IL10RA(hom p.Phe239Ser)  IL17RA(hom c.310+3G>A) | 9 |
| P119 | p.Ala181Glu | Het | - | - | - | C3 (het p.Lys155Gln) | 11 |
| P120 | p.Arg72His | Het | - | IKBKG (hom p.Leu51Phe) | - | - | 7 |
| P121 | p.Cys193Arg  p.Ser144Ter | Comp Het | - | - | - | MAP1LC3B2(het p.Asn74Ser)  NLRP2(het p.Arg441Gln) | 4 |
| P122 | p.Ala181Glu | Het | - | CHD7 (het p.His258Asn) | - | HCK (het p.N14S) | 10 |
| P123 | p.Ala181Glu | Het | - | - | - | IRF8 (het p.Ala197Val) | 7 |
| P124 | c.61+1G>T  c.61+1G>T | Hom | - | - | - | CFH(het p.Asn774Ser)  TRAF3(het p.Arg316Gln)  NLRC4(het p.R388Q) | 8 |
| P125 | p.Ala181Glu | Het | - | KMT2D (het p.Asp3419Gly)  IKBKB (het p.H7Q) | SAMD9L(het p.Arg406Ter) | GATA2(het p.Glu6Gly) | 9 |
| P126 | p.Arg72Cys | Het | - | - | - | NFAT5 (het p.Gly1352Ser)  CXCR4 (het p.L47F)  IFIH1 (het p.Glu586Gly) | 13 |
| P127 | p.Ala181Glu | Het | TCF3(het p.T531M) | NFE2L2(het p.Ser103Phe) | - | SERPING1 (het p.Ala2Val)  CASP10 (het p.Thr503Met) | 13 |
| P128 | p.Cys104Arg | Het | - | KMT2D (het p.Ile5188Val) | - | NCSTN(het p.Arg38Gly)  CASP10(het p.Lys99Glu) | 9 |
| P129 | p.Pro235ArgfsTer169 | Het | - | AK2 (comp het p.Ile223Thr/p.Ile167SerfsTer3)  KMT2A (het p.Glu2378Lys)  KMT2D (het p.Leu4498Pro) | - | CFH(hetp.Leu3Val)  POLR3A(het p.Gly701Val)  IRF3(het p.Ala277Thr)  NLRP12(het p.Gly52Ser) | 6 |
| P130 | p.Cys104Arg | Het | - | TTC7A (comp het p.Lys252Arg / p.Ser318Pro) | - | COPA (het p.Gly415Ser) | 9 |
| P131 | p.Cys104Arg | Het | - | - | - | - | 8 |
| P132 | p.Cys104Arg | Het | - | TTC7A (comp het p.Lys252Arg / p.Ser318Pro) | - | COPA (het p.Gly415Ser) | 8 |
| P133 | p.Cys104Arg | Het | - | TTC7A (comp het p.Lys252Arg / p.Ser318Pro) | - | - | 10 |
| P134 | p.Cys104Arg | Het | - | - | - | TINF2(het p.Gly25Ala)  NLRC4(het p.Gly121Val) | 9 |
| P135 | p.Ala181Glu | Het | - | KMT2A(het p.Met604Val) | - | CFHR2(het p.Arg141Ser)  CD46(het p.Ala353Val) | 13 |
| P136 | p.Ala181Glu | Het | TOP2B(het p.Val1620Ile) | - | - | TINF2(het p.Pro165Leu)  HCK(het p.H73Y) | 6 |
| P137 | p.Ala181Glu | Het | TOP2B(het p.Val1620Ile) | - | - | KMT2A(het p.Val2444Leu)  TINF2 (hom p.Pro165Leu) | 5 |
| P138 | p.Arg122Trp | Het | TCF3(het p.T531M) | KMT2D(het p.Asp3419Gly) | - | XIAP(het p.Thr470Ser)  CSF3R(het p.Ser747Asn)  NLRP2(het p.Ser626Pro)  TLR3(het p.Leu529Phe) | 3 |
| P139 | p.Arg72His | Het | TCF3(het p.Ser295del) | - | - | KMT2D (het p.Arg2635Gln)  PSTPIP1(het p.Ala362Val) | 6 |
| P140 | p.Ala181Glu | Het | - | - | - | TREX1 (het p.Glu266Gly)  NLRP2 (het p.Asp489Asn) | 8 |
| P141 | p.Arg9Gln | Het | TCF3(het p.Ala203Thr) | - | SAMD9 (het p.Glu600AspfsTer12)  ACD(het p.His289Pro) | VPS13B (comp het p.Thr1271Ser/ p.Ser2571Phe) | 14 |
| P142 | p.Gln57His | Het | - | KMT2A (het p.Arg3708His) | - | IL17RC (hom p.G76_A77insLSG)  CFH (het p.Val111Glu)  SERPING1 (het p.Ile232Leu) | 12 |
| P143 | p.Gln57His | Het | BTK (hem large deletion) | KMT2D (het p.Ser2149Pro) | SAMD9L(het p.Arg1411Gln) | NLRP12(het p.Thr260Met) | 13 |
| P144 | p.Ile87Asn | Het | - | - | - | CFH(het p.Gly286Glu)  TREX1(het p.Pro290_Ala295del)  SAMD9(het p.Gln1209Leu) | 11 |
| P145 | p.Lys188del | Het | - | - | - | - | 12 |
| P146 | p.Glu140Lys | Het | - | - | - | POLR3A(het p.Arg1245Gln)  GATA2(het p.Thr455Ala) | 10 |
| P147 | c.61+5G>A | Het | - | - | - | - | 8 |
| P148 | p.Cys104Arg | Het | - | KMT2A (het p.Pro2316Ser) | - | - | 12 |
| P149 | p.Leu69ThrfsTer12 | Het | - | - | - | CFH (het p.Asp575Val)  NLRP2 (het p.Asn853Ser)  HCK(het p.I128F)  C4A(het p.Ser1286Ala) | 5 |
| P150 | p.Pro151Leu | Het | - | KMT2D(het p.Pro3665Ala) | FANCD2(comp het p.V427Ffs*19/ p.Ala1149Val)  SAMD9L(het p.Asn1502Asp) | IFIH1(het p.Lys553Ile)  NOD2(het p.Arg791Gln) | 13 |
| P151 | p.Arg67Ser | Het | - | - | - | - | 10 |
| P152 | p.Cys104Arg | Het | - | - | - | CFTR(comp het p.Val201Met/ p.His1054Arg)  MEFV(het p.Glu230Lys)  PLCG2(het p.Pro522Arg)  CARD14(het p.Asp444Asn)  APOL1(het p.Ile171Val) | 13 |
| P153 | p.Ala181Glu | Het | - | - | - | KMT2A (het p.Ser1325Asn)  TINF2 (het p.Gly25Ala)  NLRP2 (het p.Ser626Pro)6 | 6 |
| P154 | p.Gly290Asp | Het | - | LRBA (hom p.Val737Ile)  KMT2D (het p.Pro886Thr)  FAT4(comp het p.Ser686Asn/ p.Ile897Val) | - | CFHR1(het p.Val7Ter)  LYST(comp het p.Asp2228His/ p.Asn1228Ser)  NOD2(het p.Ala1000Thr)  TICAM1(het p.Arg75Cys)  NFKBID(het c.91+2T>C)  RANBP2(het p.Asp77Glu)  IFIH1(het p.Arg598His)  TLR3(het p.Gly743Asp)  TMEM173(comp het p.Val194Leu/ p.Leu126Ile)  IFNGR1(het p.Gly170Arg) | 24 |
| P155 | p.Arg122Trp | Het | PIK3CD(het p.Thr421Ala) | IKZF2(het p.Phe531Ser)  KMT2D(het p.Thr4022Met) |  | ATAD3A(het p.Asn333His )  NLRC4(het p.Thr101Ile)  RANBP2(het p.Lys829Ile)  IFNGR1(he tp.Gly170Arg) | 9 |
| P156 | p.Arg72His | Het | - | CHD7 (het p.Leu2984Phe)  KMT2D (het p.Pro2382Ser) | - | - | 5 |
| P157 | p.Arg198His | Het | - | - | - | NOD2(hetp.Asp357Ala) | 1 |
| P158 | p.Cys172Tyr | Het | - | - | - | XIAP (hom p.Thr470Ser) | 2 |
| P159 | p.Ala181Glu | Het | - | - | - | C3 (het p.Lys155Gln) | 4 |
| P160 | p.Arg72His | Het | - | IKBKG (hom p.Leu51Phe) | - | - | 3 |
| P161 | p.Cys193Arg  p.Ser144Ter | Comp Het | - | - | - | - | 2 |
