# Supplementary material for: Re-evaluation of the contribution of TNFRSF13B variants to antibody deficiency
Source: J Hum Immun. 2025 Aug 19;1(4):e20250016. doi: 10.70962/jhi.20250016 (PMC12435966; doi:10.70962/jhi.20250016)
Supplement: Table S3 — shows the top 50 different IEI modifier gene variants (all rare variants) between 161 TACI patients and 1,241 unsolved PAD patients. [file jhi_20250016_tables3.docx]

| **Table S3-** Top 50 different IEI modifier gene variants (all rare variants) between 161 TACI patients and 1,241 unsolved PAD patients. Red color depicts significant genes. | | | | | | | | | | |
| --- | --- | --- | --- | --- | --- | --- | --- | --- | --- | --- |
| **Gene** | **TACI patients** | **Other PADs** | **p_value** | **FDR-adjusted p-value** |  | **Gene** | **TACI patients** | **Other PADs** | **p_value** | **FDR-adjusted p-value** |
| DKC1 | 4 | 0 | 9.13E-06 | 0.002188 |  | MAP1LC3B2 | 3 | 4 | 0.07327 | 0.948552 |
| JAGN1 | 12 | 16 | 9.48E-06 | 0.001514 |  | C1QA | 3 | 4 | 0.07327 | 0.92359 |
| CXCR4 | 9 | 13 | 0.000337 | 0.032314 |  | RAG2 | 9 | 29 | 0.084399 | 1.010681 |
| TET2 | 17 | 43 | 0.000794 | 0.063419 |  | STIM1 | 1 | 38 | 0.089507 | 1.045698 |
| IL10RA | 11 | 23 | 0.001864 | 0.127552 |  | IL17RE | 4 | 8 | 0.09488 | 1.082078 |
| TMEM173 | 10 | 20 | 0.002288 | 0.137021 |  | KRAS | 4 | 8 | 0.09488 | 1.056914 |
| TGFBR2 | 9 | 18 | 0.004176 | 0.181829 |  | RUNX1 | 1 | 37 | 0.097426 | 1.060618 |
| XIAP | 5 | 6 | 0.005807 | 0.231786 |  | IL2RB | 5 | 12 | 0.097477 | 1.037585 |
| RANBP2 | 32 | 125 | 0.007906 | 0.270513 |  | OAS1 | 2 | 45 | 0.117931 | 1.15284 |
| PTPRC | 1 | 62 | 0.012201 | 0.389622 |  | PIK3CG | 6 | 17 | 0.118138 | 1.109567 |
| SON | 5 | 8 | 0.02016 | 0.603543 |  | FAT4 | 33 | 162 | 0.122648 | 1.129773 |
| TLR8 | 4 | 5 | 0.020635 | 0.58142 |  | FAS | 3 | 5 | 0.12381 | 1.118958 |
| TTC7A | 20 | 75 | 0.028013 | 0.745464 |  | WAS | 1 | 34 | 0.125787 | 1.095493 |
| DIAPH1 | 1 | 50 | 0.032761 | 0.784623 |  | CFP | 1 | 34 | 0.125787 | 1.075931 |
| PTEN | 1 | 50 | 0.032761 | 0.74726 |  | C1QC | 7 | 22 | 0.129907 | 1.091675 |
| TNFRSF1A | 3 | 3 | 0.036417 | 0.792888 |  | CFTR | 29 | 140 | 0.130332 | 1.076362 |
| CASP8 | 9 | 25 | 0.036681 | 0.763922 |  | CTPS1 | 4 | 9 | 0.134236 | 1.089815 |
| HCK | 6 | 13 | 0.03812 | 0.760811 |  | BLK | 3 | 52 | 0.144061 | 1.112984 |
| POLR3A | 8 | 21 | 0.03868 | 0.741102 |  | ILK | 2 | 2 | 0.144243 | 1.096704 |
| BTK | 1 | 48 | 0.038681 | 0.712628 |  | NLRP2 | 18 | 80 | 0.148974 | 1.097821 |
| NBAS | 26 | 110 | 0.044122 | 0.782755 |  | DOCK2 | 5 | 69 | 0.155268 | 1.126868 |
| ERBB2IP | 1 | 44 | 0.054009 | 0.892082 |  | RELA | 9 | 33 | 0.160589 | 1.13121 |
| RAD51 | 2 | 1 | 0.05627 | 0.89845 |  | C4A | 2 | 41 | 0.161916 | 1.124023 |
| ATM | 14 | 161 | 0.063738 | 0.897957 |  | STK4 | 5 | 14 | 0.164403 | 1.124987 |
| NFKB1 | 7 | 19 | 0.067754 | 0.927269 |  | SLC46A1 | 5 | 14 | 0.164403 | 1.109142 |
