# Supplementary material for: Re-evaluation of the contribution of TNFRSF13B variants to antibody deficiency
Source: J Hum Immun. 2025 Aug 19;1(4):e20250016. doi: 10.70962/jhi.20250016 (PMC12435966; doi:10.70962/jhi.20250016)
Supplement: Table S4 — shows the top 50 different non-IEI modifier gene mutations (all rare variants) between 161 TACI patients and 1,241 unsolved PAD patients. [file jhi_20250016_tables4.docx]

| **Table S4-** Top 50 different non-IEI modifier gene mutations (all rare variants) between 161 TACI patients and 1,241 unsolved PAD patients. | | | | | | | | | | |
| --- | --- | --- | --- | --- | --- | --- | --- | --- | --- | --- |
| **Gene** | **TACI patients** | **Other PADs** | **p_value** | **FDR-adjusted p-value** |  | **Gene** | **TACI patients** | **Other PADs** | **p_value** | **FDR-adjusted p-value** |
| PRAMEF5 | 64 | 64 | 4.36E-34 | 3.13E-30 |  | MST1L | 69 | 193 | 5.90E-10 | 2.82E-07 |
| AGAP5 | 53 | 57 | 1.01E-26 | 4.83E-23 |  | USP17L18 | 31 | 54 | 6.43E-10 | 2.97E-07 |
| FAM86B1 | 48 | 47 | 3.02E-26 | 1.08E-22 |  | RNU6-1240P | 28 | 45 | 6.48E-10 | 2.90E-07 |
| OR1D4 | 45 | 50 | 3.35E-22 | 9.60E-19 |  | RNU6-1268P | 36 | 71 | 1.02E-09 | 4.42E-07 |
| MUC19 | 588 | 2569 | 2.07E-19 | 4.94E-16 |  | MUC16 | 215 | 891 | 1.10E-09 | 4.63E-07 |
| CLEC18C | 39 | 44 | 4.12E-19 | 8.44E-16 |  | DOCK11 | 31 | 57 | 2.71E-09 | 1.11E-06 |
| KRT18 | 27 | 19 | 5.61E-19 | 1.01E-15 |  | ZNF92 | 14 | 12 | 5.41E-09 | 2.15E-06 |
| MUC12 | 179 | 556 | 6.27E-19 | 9.98E-16 |  | STAG3 | 26 | 43 | 5.50E-09 | 2.13E-06 |
| GAGE12F | 17 | 4 | 8.66E-19 | 1.24E-15 |  | MUC5B | 209 | 881 | 7.01E-09 | 2.65E-06 |
| NES | 61 | 111 | 1.64E-17 | 2.14E-14 |  | AGAP3 | 28 | 51 | 1.38E-08 | 4.95E-06 |
| MUC6 | 121 | 337 | 1.09E-16 | 1.21E-13 |  | VEGFC | 23 | 38 | 4.60E-08 | 1.61E-05 |
| KIAA1522 | 51 | 86 | 2.74E-16 | 2.81E-13 |  | ZNF302 | 50 | 721 | 4.82E-08 | 1.65E-05 |
| ANKRD20A4 | 60 | 120 | 3.47E-15 | 3.32E-12 |  | FOXD4L3 | 18 | 24 | 5.00E-08 | 1.67E-05 |
| NBPF1 | 108 | 310 | 3.87E-14 | 3.46E-11 |  | SPDYE6 | 19 | 27 | 6.15E-08 | 2.00E-05 |
| RAB41 | 14 | 5 | 9.28E-14 | 7.82E-11 |  | OR1D5 | 15 | 17 | 7.98E-08 | 2.54E-05 |
| FAM86B2 | 38 | 59 | 1.27E-13 | 9.56E-11 |  | FRG1 | 56 | 162 | 8.82E-08 | 2.75E-05 |
| PRSS3 | 48 | 91 | 2.73E-13 | 1.96E-10 |  | GOLGA6B | 22 | 37 | 1.33E-07 | 4.05E-05 |
| GAGE2A | 22 | 20 | 3.47E-13 | 2.37E-10 |  | NPC1L1 | 42 | 109 | 2.14E-07 | 6.38E-05 |
| POTEB2 | 23 | 23 | 8.25E-13 | 5.37E-10 |  | TTK | 21 | 35 | 2.27E-07 | 6.64E-05 |
| KLC4 | 31 | 44 | 2.08E-12 | 1.24E-09 |  | RIMBP3C | 8 | 4 | 4.51E-07 | 0.000129 |
| FLG | 155 | 557 | 1.99E-11 | 1.14E-08 |  | POTEG | 37 | 93 | 5.37E-07 | 0.000151 |
| VCX3B | 47 | 104 | 1.66E-10 | 9.18E-08 |  | GPR42 | 17 | 25 | 5.88E-07 | 0.000162 |
| CEP89 | 27 | 40 | 1.84E-10 | 9.79E-08 |  | SCARB1 | 11 | 10 | 6.27E-07 | 0.00017 |
| PRAMEF2 | 52 | 123 | 2.23E-10 | 1.14E-07 |  | CTPS2 | 11 | 10 | 6.27E-07 | 0.000166 |
| C2CD2 | 25 | 36 | 4.88E-10 | 2.41E-07 |  | PRAMEF4 | 30 | 68 | 7.63E-07 | 0.000195 |
