# Supplementary material for: Re-evaluation of the contribution of TNFRSF13B variants to antibody deficiency
Source: J Hum Immun. 2025 Aug 19;1(4):e20250016. doi: 10.70962/jhi.20250016 (PMC12435966; doi:10.70962/jhi.20250016)
Supplement: Table S5 — shows the main modifier gene polymorphism in the significantly enriched pathways between 161 TACI patients and 1,241 unsolved PAD patients. [file jhi_20250016_tables5.docx]

| **Table S5-** Main modifier gene polymorphism in the significantly enriched pathways between 161 TACI patients and 1,241 unsolved PAD patients. Red color depicts significant preventive polymorphisms. | | | | | | | | | | |
| --- | --- | --- | --- | --- | --- | --- | --- | --- | --- | --- |
| **SNP** | **Associated gene** | **%in TACI patients** | **% in Other PADs** | **p_value** |  | **SNP** | **Associated gene** | **%in**  **TACI patients** | **%in Other PADs** | **p_value** |
| rss2688539 | MUC20 | 15.62 | 0.98 | 2.91E-39 |  | rs369371347 | MUC6 | 19.79 | 5.39 | 1.77E-14 |
| rs200702634 | MUC12 | 11.46 | 0.74 | 9.45E-29 |  | rs2303425 | MSH2 | 18.23 | 4.80 | 5.79E-14 |
| rs201905745 | MUC12 | 11.46 | 0.74 | 9.45E-29 |  | rs1182136 | CARD11 | 73.96 | 45.69 | 6.59E-14 |
| rs760572685 | MUC12 | 5.73 | 0.00 | 2.28E-27 |  | rs376067175 | MUC6 | 14.06 | 3.04 | 8.45E-14 |
| rs199931267 | MUC12 | 6.77 | 0.10 | 2.79E-27 |  | rs2274892 | TNFRSF13B | 15.62 | 43.04 | 1.44E-13 |
| rs201665808 | MUC12 | 6.25 | 0.05 | 3.63E-27 |  | rs12446127 | PLCG2 | 71.35 | 43.77 | 2.30E-13 |
| rs199764232 | MUC12 | 10.42 | 0.69 | 6.68E-26 |  | rs10172036 | ICOS | 24.48 | 52.11 | 2.48E-13 |
| rs200820773 | MUC12 | 7.29 | 0.25 | 2.97E-24 |  | rs79916386 | MUC19 | 6.25 | 0.59 | 3.54E-13 |
| rs78879019 | MUC16 | 12.50 | 1.47 | 1.92E-21 |  | rs7711953 | SPINK5 | 22.40 | 49.66 | 4.72E-13 |
| rs3771274 | MSH2 | 6.25 | 0.20 | 1.98E-21 |  | rs77879173 | MUC4 | 7.29 | 0.88 | 9.18E-13 |
| rs767254545 | MUC16 | 17.71 | 3.09 | 2.12E-21 |  | rs12445580 | PLCG2 | 67.19 | 40.64 | 1.20E-12 |
| rs76605488 | MUC19 | 5.73 | 0.15 | 7.52E-21 |  | rs200706535 | MUC20 | 15.62 | 3.97 | 1.23E-12 |
| rs201997835 | MUC6 | 12.50 | 1.57 | 2.06E-20 |  | rs373360588 | MUC6 | 16.15 | 4.22 | 1.33E-12 |
| rs12998837 | MSH2 | 18.23 | 3.63 | 2.84E-19 |  | rs12812028 | MUC19 | 14.58 | 3.58 | 2.32E-12 |
| rs200583897 | MUC12 | 4.69 | 0.10 | 3.90E-18 |  | rs200869475 | MUC12 | 8.33 | 1.23 | 2.35E-12 |
| rs199726057 | MUC12 | 4.17 | 0.05 | 7.48E-18 |  | rs79198757 | MUC6 | 13.54 | 3.14 | 2.54E-12 |
| rs200182709 | MUC20 | 7.29 | 0.54 | 1.90E-17 |  | rs527248643 | MUC6 | 14.06 | 3.38 | 3.09E-12 |
| rs199522572 | MUC12 | 5.21 | 0.20 | 4.10E-17 |  | rs3136284 | MSH6 | 3.65 | 0.15 | 3.92E-12 |
| rs2306571 | PRKCD | 69.27 | 38.68 | 1.86E-16 |  | rs199800263 | MUC4 | 3.65 | 0.15 | 3.92E-12 |
| rs75583821 | MUC4 | 4.69 | 0.15 | 1.95E-16 |  | rs1422991 | SPINK5 | 22.92 | 49.02 | 4.20E-12 |
| rs10751676 | MUC6 | 20.31 | 5.20 | 4.48E-16 |  | rs11958432 | SPINK5 | 22.40 | 48.43 | 4.58E-12 |
| rs1289384896 | MUC19 | 5.21 | 0.25 | 8.47E-16 |  | rs2303061 | SPINK5 | 22.92 | 48.97 | 4.60E-12 |
| rs1221710360 | MUC19 | 5.21 | 0.25 | 8.47E-16 |  | rs2303062 | SPINK5 | 22.92 | 48.97 | 4.60E-12 |
| rs4509369 | CHD7 | 16.67 | 46.18 | 3.21E-15 |  | rs2303068 | SPINK5 | 22.92 | 48.97 | 4.60E-12 |
| rs3764926 | SPINK5 | 75.52 | 45.93 | 4.40E-15 |  | rs1800937 | MSH6 | 13.02 | 2.99 | 5.07E-12 |
| rs775515818 | MUC6 | 16.15 | 3.63 | 4.80E-15 |  | rs548701018 | MUC6 | 13.54 | 3.28 | 1.05E-11 |
| rs200229903 | MUC12 | 5.21 | 0.29 | 1.20E-14 |  |  |  |  |  |  |
